# Nanometric Faujasite (FAU) Zeolite Ion‐Exchanged With Metal Ions for Hemostatic and Antimicrobial Applications: A Thromboelastographic and Microbiological Study

**DOI:** 10.1002/asia.70755

**Published:** 2026-04-29

**Authors:** Guilherme de Paula Guarnieri, Juliana Bergamasco Laurenti, Beatriz Crespo, Edivandra Buzato Silva, Taiza Maschio‐Lima, Vilson Serafim Júnior, Eny Maria Goloni‐Bertollo, Moacir Fernandes de Godoy, Margarete Teresa Gottardo de Almeida, José Geraldo Nery

**Affiliations:** ^1^ Department of Physics São Paulo State University São José do Rio Preto São Paulo Brazil; ^2^ Laboratory of Microbiology Faculty of Medicine of São José Do Rio Preto São José do Rio Preto São Paulo Brazil; ^3^ Genetics and Molecular Biology Research Unit Faculty of Medicine of São José Do Rio Preto São José do Rio Preto São Paulo Brazil; ^4^ Department of Cardiology and Cardiovascular Surgery Faculty of Medicine of São José Do Rio Preto São José do Rio Preto São Paulo Brazil

## Abstract

Integrating antimicrobial functionality with the intrinsic clot‐promoting properties of zeolites represents a promising strategy for next‐generation wound‐care materials, where rapid hemorrhage control must be coupled with infection prevention. Here, we investigate nanocrystalline Faujasite (NanoFAU) and its ion‐exchanged derivatives to elucidate how extra‐framework cation identity governs the balance between hemostatic and antimicrobial performance. NanoFAU samples exchanged with Ag^+^, Ba^2^^+^, Ca^2^^+^, and Mg^2^^+^ were comprehensively characterized by XRD, SEM/HRTEM/EDS, AFM, BET surface area analysis, ^2^ ^9^Si MAS NMR and FT‐IR spectroscopy, confirming preservation of the FAU framework and successful cation substitution. All ion‐exchanged materials exhibited isoelectric points below physiological blood pH, consistent with negatively charged, procoagulant surfaces. Thromboelastography revealed that Ca^2^^+^‐exchanged NanoFAU (NanoFAU‐Ca) achieved the most pronounced hemostatic response (*R* = 1.1 min; *K* = 1.2 min; MA = 60.3 mm), whereas Ag^+^‐exchanged NanoFAU (NanoFAU‐Ag) provided strong antimicrobial activity against *Staphylococcus aureus* (ATCC 25923) and *Candida albicans* (ATCC 90028), displaying bactericidal and fungistatic effects, respectively. Importantly, NanoFAU‐Ag maintained cell viability above the ISO 10993‐5 cytotoxicity threshold in HaCaT keratinocytes. These findings demonstrate that ion exchange enables rational tuning of NanoFAU to achieve complementary hemostatic and antimicrobial functionalities while preserving cytocompatibility, establishing NanoFAU as a versatile inorganic platform for multifunctional wound management and supporting future in vivo evaluation.

## Graphical abstract

Faujasite nanozeolite (NanoFAU) and its ion‐exchanged forms (Ag^+^, Ba^2+^, Ca^2+^, Mg^2+^) were synthesized and characterized to assess their biomedical potential. NanoFAU‐Ca showed superior hemostatic activity, while NanoFAU‐Ag exhibited strong antimicrobial effects against *S. aureus* and *C. albicans*. These findings highlight NanoFAU derivatives as multifunctional materials for hemorrhage control and infection prevention.

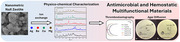

## 1 Introduction

Zeolites are crystalline aluminosilicates composed of periodic three‐dimensional frameworks of cages and channels with molecular‐scale dimensions. Owing to their well‐defined porosity, high surface area, and tunable framework chemistry, zeolites are extensively employed in industrial applications as catalysts, adsorbents, and ion exchangers. In parallel, zeolite thin films and nanostructured forms have attracted considerable interest for advanced technologies, including selective membranes, chemical sensors, and microelectronic devices [1, 2, 3, 4]. Structurally, zeolites are constructed from corner‐sharing [SiO_4_] and [AlO_4_] tetrahedra interconnected via oxygen bridges, generating extended nanoporous networks with large internal voids and interconnected channels. This distinctive topology underpins their high sorption capacity and ion‐exchange versatility [5, 6].

In recent years, zeolites have also gained increasing attention in the biomedical field, motivated by their intrinsic biocompatibility, structural stability under physiological conditions, and capacity to host and release functional species in a controlled manner [6, 7, 8]. Among these applications, dehydrated zeolite powders have been widely explored as topical hemostatic agents for the rapid management of severe hemorrhage. A notable example is QuikClot, a zeolite‐based material deployed during the Iraq War in 2003 for emergency bleeding control [9]. Acting as molecular sieves, zeolites rapidly adsorb water from blood at wound sites, thereby concentrating clotting factors, platelets, and erythrocytes and accelerating activation of the coagulation cascade. This effect can be further modulated by the release of biologically relevant cations, such as Ca^2^^+^, which play a central role in multiple steps of the coagulation process [7, 10, 11, 12].

Beyond water adsorption‐driven mechanisms, accumulating evidence suggests that zeolites may actively participate in coagulation at the molecular level. Owing to their negatively charged surfaces—originating from framework Al substitution and deprotonated silanol groups with low p*K*_a_ values [13]—zeolites can function as so‐called “inorganic platelets” upon contact with whole blood at physiological pH (∼7.4). These negatively charged interfaces promote the assembly of prothrombinase complexes in a manner analogous to the phospholipid surfaces of activated platelets, enhancing the conversion of prothrombin to thrombin and accelerating fibrin formation [14, 15].

A key advantage of zeolites lies in the tunability of their physicochemical properties through framework composition and post‐synthetic modification. Isomorphic substitution of Si^4^^+^ by Al^3^^+^ within the tetrahedral framework generates a permanent negative charge that is compensated by extra‐framework cations. Although these cations are an integral part of the crystallographic structure, they can be readily replaced by alternative ions via ion‐exchange processes [6]. While ion exchange is generally straightforward from a synthetic perspective, it can profoundly alter surface chemistry, hydration behavior, and biological response, often in complex and nonlinear ways. For example, exchange of zeolite LTA‐5A with Ag^+^ not only imparts pronounced antimicrobial activity but also significantly reduces the enthalpy of hydration—from approximately 680 to 420 J g^−^^1^—thereby mitigating excessive heat release during water adsorption, a critical safety consideration for topical hemostatic applications [16]. Although direct temperature‐rise measurements were not performed in the present study, prior calorimetric analysis and the altered hydration dynamics of nanometric zeolites support the expectation that nanoscale systems may further attenuate localized thermal effects due to enhanced heat dissipation and modified water adsorption behavior [16].

In biomaterials research, ion exchange has been widely exploited to introduce antimicrobial functionality into inorganic matrices through incorporation of metal ions such as Ag^+^ and Cu^2^^+^, which can be released in a controlled fashion upon exposure to physiological fluids [17, 18, 19, 20, 21]. Zeolite‐based antimicrobial materials have demonstrated efficacy against a broad spectrum of clinically relevant microorganisms, including *Staphylococcus aureus*, *Escherichia coli*, and *Candida albicans* [17, 22, 23, 24, 25]. The renewed interest in metal‐based antimicrobials is driven by the accelerating global crisis of antibiotic resistance, as metal ions typically exert antimicrobial activity through multiple, nonspecific mechanisms—such as membrane disruption, protein inactivation, and oxidative stress induction—therefore reducing the likelihood of resistance development compared to conventional antibiotics that target specific biochemical pathways [26, 27].

However, the simultaneous integration of hemostatic and antimicrobial functionalities within a single inorganic platform presents significant physicochemical and biological challenges. Metal ions that confer antimicrobial activity—particularly Ag^+^—can interfere with coagulation processes by interacting with plasma proteins, altering fibrin polymerization, or perturbing platelet–fibrin interactions. Moreover, antimicrobial ion release must be sufficient to inhibit microbial growth while remaining below cytotoxic thresholds for mammalian cells. Changes in surface charge, hydration behavior, and ion mobility introduced during ion exchange may therefore enhance one biological function while inadvertently suppressing another. Achieving a balanced multifunctional profile thus requires careful control of ionic identity and framework chemistry, rather than simple incorporation of antimicrobial species.

In this work, we investigate the concurrent hemostatic and antimicrobial properties of nanocrystalline Faujasite (FAU) zeolites before and after ion exchange with Ag^+^, Ba^2^^+^, Ca^2^^+^, and Mg^2^^+^. These cations were selected based on their documented roles in coagulation pathways or antimicrobial activity [15, 28, 29, 30]. Hemostatic performance was quantitatively assessed by thromboelastography (TEG), while antimicrobial efficacy was evaluated using minimum inhibitory concentration (MIC) assays against *S. aureus* and *C. albicans*, two clinically relevant pathogens frequently associated with wound infections. The results presented herein provide a structure–function framework for understanding how ion exchange modulates the dual biological activity of FAU nanozeolites and establish a foundation for future in vivo studies aimed at evaluating their performance under physiological conditions.

## 2 Experimental Section

### 2.1 Synthesis and Physicochemical Characterization of the Nanocrystalline Zeolites

#### 2.1.1 Synthesis of Nanocrystalline Faujasite (NanoFAU)

NanoFAU zeolite was synthesized following the procedure reported by Zhan et al. [31], with minor modifications. In a typical synthesis, sodium hydroxide was first dissolved in deionized water under continuous stirring. Sodium aluminate was then added gradually to the alkaline solution, and the mixture was stirred at room temperature for 15 min until a clear and homogeneous solution was obtained. Subsequently, fumed silica was introduced as the silica source, yielding a viscous suspension that was maintained under constant agitation at 60°C for 48 h to promote nucleation and crystallization of the FAU framework. After crystallization, the solid product was recovered by centrifugation at 13,400 rpm, thoroughly washed with deionized water until neutral pH was achieved, and dried in a convection oven at 40°C for 16 h. The overall molar composition of the synthesis gel was 5.5 Na_2_O:1 Al_2_O_3_:4 SiO_2_:190 H_2_O.

#### 2.1.2 Ion Exchange With Metal Ions

Ion exchange of the as‐synthesized NanoFAU was carried out using aqueous solutions of copper nitrate, magnesium chloride, and silver nitrate (0.5 M). The procedure was adapted from Vasconcellos et al. [32]. In a typical experiment, 1.0 g of NanoFAU was dispersed in 30 mL of the corresponding metal salt solution in a Teflon‐lined vessel and maintained at 90°C for 12 h under gentle magnetic stirring to promote cation exchange. After completion of the exchange process, the solid products were recovered by centrifugation at 13,400 rpm, washed repeatedly with deionized water (three cycles) to remove non‐incorporated ions, and dried in a convection oven at 40°C for 16 h. The resulting materials are hereafter denoted as metal‐exchanged NanoFAU.

#### 2.1.3 x‐Ray Diffraction (XRD)

Powder XRD patterns of the synthesized materials were recorded on a Rigaku MiniFlex II diffractometer (Rigaku Corporation, Tokyo, Japan) operating in Bragg–Brentano reflection geometry with a flat‐plate sample holder. The instrument was equipped with a Cu Kα radiation source (*λ* = 1.5418 Å) and operated at 40 kV and 15 mA. A graphite monochromator was employed to ensure monochromatic radiation. Diffraction data were collected over a 2*θ* range of 3°–50°, using a step size of 0.02° (2*θ*) and a counting time of 10 s per step.

#### 2.1.4 Scanning/Transmission Electron Microscopy (SEM and TEM) and Energy‐Dispersive x‐Ray Spectroscopy (EDS)

The morphology and particle size distribution of the zeolite samples were examined by SEM using a Philips XL30 FEG microscope operated at accelerating voltages between 5 and 25 kV. Prior to imaging, the samples were sputter‐coated with a thin gold layer to minimize surface charging. SEM analyses were carried out at the Structural Characterization Laboratory of the Federal University of São Carlos (LCE/DEMa/UFSCar), São Carlos, Brazil. Elemental composition and ion‐exchange efficiency were evaluated by EDS using an EVEX EDS detector coupled to the SEM system at LCE/DEMa/UFSCar. For quantitative analysis, the relative atomic percentages obtained by EDS were used.

High‐resolution transmission electron microscopy (HRTEM) images were acquired using an FEI Tecnai G2 F20 microscope operated at 200 kV at the Structural Characterization Laboratory of the Federal University of São Carlos (LCE/DEMa/UFSCar), São Carlos, Brazil. The oxide compositions reported in Table 1 were calculated from the atomic percentages obtained by EDS. Considering that the concentration of charge‐compensating extra‐framework cations is stoichiometrically related to the number of framework [AlO_4_]^−^ units, the compositions were normalized with respect to Al_2_O_3_. This normalization enabled quantitative estimation of the ion‐exchange degree by comparing the Na^+^ content of the exchanged samples with that of the parent NanoFAU. The exchange degree was thus expressed as the percentage of Na^+^ removed relative to the original material.

**TABLE 1:** Compositional data extracted from the EDS spectra. The oxide formulas were normalized to Al_2_O_3_. Exchanged Na^+^ was determined from the normalized oxide formulas and represents the proportion of Na^+^ ions removed in the ion‐exchanged samples relative to the original NanoFAU.

	Na	Al	Si	Ca	Mg	Ba	Ag	Oxide formula	Exchanged Na^+^
NanoFAU	35.5	26.7	37.8	—	—	—	—	1.0 Al_2_O_3_:2.8 SiO_2_:1.3 Na_2_O	—
NanoFAU‐Ca	7.0	34.2	45.6	13.1	—	—	—	1.0 Al_2_O_3_:2.7 SiO_2_:0.2 Na_2_O:0.8 CaO	84.6%
NanoFAU‐Mg	13.8	29.0	39.3	—	17.9	—	—	1.0 Al_2_O_3_:2.7 SiO_2_:0.5 Na_2_O:1.2 MgO	61.5%
NanoFAU‐Ba	4.3	34.8	46.1	—	—	14.8	—	1.0 Al_2_O_3_:2.6 SiO_2_:0.1 Na_2_O: 0.9 BaO	92.3%
NanoFAU‐Ag	—	29.0	38.0	—	—	—	33.0	1.0 Al_2_O_3_:2.6 SiO_2_:0.0 Na_2_O:1.1 Ag_2_O	100.0%

#### 2.1.5 Atomic Force Microscopy (AFM)

AFM analyses were performed using an NX‐10 scanning probe microscope (Park Systems) operated in tapping mode under ambient conditions. Measurements were conducted using a PPP‐CNDH silicon cantilever (Park Systems) with a nominal resonance frequency of approximately 320 kHz and a spring constant of 42 N m^−^^1^. For sample preparation, the zeolite powders were dispersed in distilled water, drop‐cast onto freshly cleaved mica substrates, and dried under high vacuum prior to analysis. AFM images were processed and quantitatively analyzed using Gwyddion software (32‐bit version). All AFM measurements were carried out at the National Nanotechnology Laboratory (LNNano), Brazilian Center for Research in Energy and Materials (CNPEM), Campinas, Brazil.

#### 2.1.6 Determination of the Isoelectric Point (IEP) by Zeta Potential Analysis

The IEP of the synthesized nanozeolites was determined from zeta potential measurements using a Malvern Zetasizer Nano ZS instrument. The experimental protocol was adapted from Ostomel et al. [33]. This analysis was performed to evaluate the surface charge behavior of the zeolitic materials and to infer their procoagulant or anticoagulant tendencies based on the pH value corresponding to the IEP. Due to the colloidal complexity and heterogeneous composition of whole blood, direct in situ measurements of zeta potential are not feasible. Consequently, surface charge characteristics were assessed indirectly through electrophoretic mobility measurements in aqueous media, which provide a well‐established and widely accepted proxy for probing the electrostatic properties of solid–liquid interfaces under controlled conditions [33]. For zeolitic systems in particular, zeta potential analysis has been shown to be sensitive to framework composition, extra‐framework cation identity, and pH, thereby enabling meaningful comparison of surface charge behavior across different ion‐exchanged materials [34]. To approximate physiological ionic conditions, measurements were conducted in the presence of CaCl_2_ as a supporting electrolyte, reproducing calcium ion concentrations representative of blood serum [35]. The presence of divalent cations is especially relevant, as Ca^2^^+^ plays a central role in both coagulation cascades and surface‐mediated protein adsorption processes: previous studies have demonstrated that calcium‐containing zeolites exhibit distinct surface charge characteristics and protein interaction patterns that can modulate contact activation and thrombin generation [15, 36, 37, 38]. For each analysis, approximately 2 mg of zeolite sample was dispersed in 60 mL of a 0.03 mol L^−^^1^ aqueous CaCl_2_ solution and sonicated for 10 min to ensure homogeneous dispersion. Zeta potential values were recorded as a function of pH over the range of 2–12. pH adjustments were performed by titration with standardized 0.25 mol L^−^^1^ HCl and NaOH solutions. The IEP was determined as the pH at which the zeta potential crossed zero.

#### 2.1.7 Textural Characterization by N_2_ Adsorption–Desorption

Nitrogen adsorption–desorption measurements were carried out to evaluate the textural properties of the synthesized zeolites, including specific surface area, total pore volume, micropore volume, and pore size distribution. Adsorption isotherms were recorded at 77 K (liquid nitrogen temperature) using a Quantachrome Nova 2000e surface area analyzer. Prior to analysis, the samples were degassed under vacuum at 150°C for 4 h to remove physisorbed moisture and residual surface species. Degassing conditions were selected to ensure complete desorption of weakly bound species while preserving the structural integrity of the FAU framework. The specific surface area was calculated using the Brunauer–Emmett–Teller (BET) method within the relative pressure (*P*/*P*_0_) range consistent with BET applicability criteria. The total pore volume was determined from the amount of nitrogen adsorbed at *P*/*P*_0_ ≈ 0.99. Micropore volume was estimated using the Saito–Foley (SF) method, while mesopore volume and pore size distribution were evaluated using the Barrett–Joyner–Halenda (BJH) model applied to the adsorption branch of the isotherm. Textural parameters were calculated in accordance with IUPAC recommendations for gas physisorption analysis [39, 40]. All measurements were performed at the Center for Ceramic Coatings (CRC), Federal University of São Carlos (UFSCar), São Carlos, Brazil.

#### 2.1.8 ^2^ ^9^Si Magic Angle Spinning (MAS) Nuclear Magnetic Resonance (^2^ ^9^Si MAS NMR)

Solid‐state nuclear magnetic resonance (NMR) spectroscopy was employed to investigate the local silicon environments in the synthesized zeolitic materials. ^2^ ^9^Si MAS NMR experiments were performed on a Bruker Avance III 400 spectrometer operating at a magnetic field strength of 9.4 T, corresponding to a Larmor frequency of 79.5 MHz for ^2^ ^9^Si nuclei. The spectrometer was equipped with a 4 mm double‐resonance MAS probe suitable for broadband heteronuclear experiments. Samples were packed into 4 mm zirconia rotors fitted with Kel‐F caps and spun at a MAS frequency of 10 kHz under ambient conditions. The MAS frequency and angle were calibrated using the ^7^ ^9^Br resonance of a KBr standard, and magnetic field homogeneity was optimized by minimizing the linewidth of the spinning sidebands. When required, the probe configuration allowed stable spinning at frequencies up to 15 kHz. Time‐domain signals were Fourier transformed into frequency‐domain spectra using SpinWorks software. Owing to the high operational costs and significant spectrometer time associated with solid‐state NMR measurements, ^2^ ^9^Si MAS NMR experiments were performed selectively on representative samples rather than on the complete sample set.

## 3 Coagulation Evaluation

### 3.1 Thromboelastography (TEG)

The coagulation performance of the zeolitic materials was evaluated by TEG using a Haemoscope TEG 5000 thrombelastograph. TEG provides a dynamic and quantitative assessment of the viscoelastic evolution of whole blood during clot initiation, propagation, and stabilization. The method enables extraction of key kinetic and mechanical parameters of the coagulation cascade, including the reaction time (*R*), corresponding to the onset of fibrin formation; the clot formation time (*K*); the *α*‐angle, reflecting the rate of fibrin polymerization and clot strengthening; and the maximum amplitude (MA), which represents the ultimate clot strength determined primarily by platelet–fibrin interactions. Citrated whole blood was employed to prevent premature coagulation. For each assay, a 20 µL aliquot of an aqueous 0.2 M CaCl_2_ solution (for recalcification) was added into the TEG sample cup. Separately, 340 µL of citrated whole blood was mixed with 0.5 mg of each zeolitic material. The mixture was then directly introduced into the TEG sample cup containing the 0.2 M CaCl_2_ solution. Measurements were conducted under standard operating conditions for whole‐blood TEG analysis. Each assay was performed in triplicate and repeated across three independent experimental runs to ensure reproducibility and statistical robustness. The thromboelastograph continuously recorded viscoelastic changes throughout clot formation, producing characteristic TEG tracings that were used for quantitative comparison of clot initiation kinetics and mechanical stability among the different zeolitic formulations. All procedures involving human blood samples were conducted in accordance with established ethical standards and were approved by the Research Ethics Committee of the São José do Rio Preto Medical School (FAMERP) under protocol number CAAE 48358215.9.0000.5415.

### 3.2 Phase‐Contrast Microscopy

Phase‐contrast optical microscopy was employed to qualitatively assess the interaction between the zeolitic materials and whole blood, with particular emphasis on fibrin network formation and cellular aggregation phenomena. Imaging was performed using an Olympus BX60 optical microscope operated in phase‐contrast mode. For each experiment, 170 µL of citrated whole blood (collected in tubes containing 4% *v*/*v* sodium citrate as anticoagulant) was deposited onto a clean glass microscope slide. Coagulation was reinitiated by the addition of 10 µL of a 0.2 mol L^−^^1^ CaCl_2_ saline solution, restoring calcium ions previously chelated by citrate and reactivating calcium‐dependent steps of the coagulation cascade. Subsequently, 0.25 mg of the respective zeolite material was introduced into the recalcified blood sample and gently mixed to ensure homogeneous contact. The prepared samples were immediately examined under phase‐contrast illumination to monitor the early stages of clot development. Particular attention was given to fibrin strand formation, platelet and erythrocyte aggregation, and the spatial organization of blood components in proximity to the zeolitic particles. Phase‐contrast microscopy was selected as it enables visualization of unstained biological specimens, thereby preserving native clot morphology and minimizing sample perturbation. All microscopy analyses were performed at the Multiuser Center for Microscopy and Microanalysis, IBILCE/UNESP, São José do Rio Preto, Brazil.

## 4 Antimicrobial Activity of Nanozeolites

### 4.1 MIC Determination Against ATCC Strains

The antimicrobial activity of NanoFAU and its ion‐exchanged derivatives with silver and magnesium (NanoFAU‐Ag and NanoFAU‐Mg) was evaluated against *S. aureus* (ATCC 25923) and *C. albicans* (ATCC 90028). MIC assays were performed using the broth microdilution method in sterile 96‐well microplates, following the recommendations of the Clinical and Laboratory Standards Institute (CLSI) guideline M27‐S4 [41]. All experiments were conducted in triplicate to ensure reproducibility. For each material, aqueous suspensions were prepared at three concentrations (0.1, 0.01, and 0.001 g), followed by sonication for 30 min to ensure homogeneous dispersion of the zeolitic particles. Aliquots (100 µL) of each suspension were transferred to individual wells, and RPMI 1640 culture medium (Sigma) was added to achieve the final assay volume. Each row of the microplate corresponded to a single zeolite concentration and formulation (NanoFAU‐Ag or NanoFAU‐Mg), tested in triplicate, and included appropriate sterility controls (medium only) and growth controls (inoculated medium without zeolite). Microbial inocula were prepared from fresh 24 h cultures grown at 36.5°C and standardized to the turbidity of a 1.0 McFarland standard. Subsequently, 100 µL of the standardized inoculum was added to each well, and the plates were incubated at 36.5°C for 24 h under aerobic conditions. Following incubation, microbial metabolic activity was assessed by the addition of 20 µL of a 2,3,5‐triphenyltetrazolium chloride (TTC) solution (0.1 g dissolved in 5 mL sterile distilled water) to each well as a redox indicator. Colorimetric changes were visually monitored to evaluate cell viability. To further confirm microbial inhibition or survival, aliquots from each well were subcultured onto Mueller–Hinton agar plates (Sigma) and incubated at 36.5°C for an additional 24 h.

### 4.2 MIC of NanoFAU and NanoFAU‐Ag Against ATCC Strains

The antimicrobial activity of NanoFAU and its silver ion‐exchanged derivative (NanoFAU‐Ag) was evaluated against *S. aureus* (ATCC 25923) and *C. albicans* (ATCC 90028). MIC assays were performed in sterile 96‐well microplates using the broth microdilution method in accordance with the CLSI guideline M27‐S4 [41]. All experiments were conducted in triplicate. Microbial inocula were prepared from fresh 24 h cultures grown at 36.5°C and adjusted to a turbidity equivalent to a 1.0 McFarland standard. The standardized suspensions were subsequently diluted in RPMI 1640 culture medium to obtain the working inoculum concentration recommended for broth microdilution assays. Serial two‐fold dilutions of NanoFAU and NanoFAU‐Ag were prepared in RPMI medium, resulting in final concentration ranges of 500–3.90 mg mL^−^^1^ for NanoFAU and 1.00–0.007 mg mL^−^^1^ for NanoFAU‐Ag. In each well, 100 µL of the zeolite suspension was combined with 100 µL of the standardized microbial inoculum, yielding a total reaction volume of 200 µL per well. Microplates were incubated at 36.5°C for 24 h under aerobic conditions. Following incubation, microbial metabolic activity was assessed by adding 20 µL of a TTC solution (0.1 g dissolved in 5 mL sterile distilled water) to each well as a redox indicator. The absence of color development was interpreted as inhibition of microbial metabolic activity. To confirm growth inhibition, aliquots from each well were subcultured onto Mueller–Hinton agar plates and incubated for an additional 24 h at 36.5°C.

### 4.3 Agar Diffusion Assay for Zeolitic Inhibition of *S. aureus* and *C. albicans*

The antimicrobial activity of selected ion‐exchanged zeolites was further assessed using the agar well diffusion method as a complementary qualitative assay. Suspensions of NaFAU‐Mg and NaFAU‐Ag were prepared in sterile aqueous medium at a concentration of 0.4 mg mL^−^^1^. Microbial inocula of *S. aureus* (ATCC 25923) and *C. albicans* (ATCC 90028) were obtained from fresh 24 h cultures and adjusted to a turbidity equivalent to a 1.0 McFarland standard. The standardized inocula were uniformly spread onto Mueller–Hinton agar plates using the spread‐plate technique with a Drigalski loop to ensure homogeneous bacterial or fungal lawns. Wells were then aseptically cut into the agar surface, and defined volumes of the zeolite suspensions were introduced into each well. The plates were incubated at 36.5°C for 24 h under aerobic conditions, after which the diameters of the inhibition zones were measured. In a second set of experiments, NaFAU, NaFAU‐Ca, NaFAU‐Ag, and a physical mixture of NaFAU‐Ca and NaFAU‐Ag were evaluated against *S. aureus* at zeolite concentrations of 0.4 and 0.8 mg mL^−^^1^. Sample preparation, inoculum standardization, agar inoculation, incubation, and inhibition zone measurements were performed following the same procedure described above. It is noted that, for insoluble particulate materials such as zeolites, inhibition zones in agar diffusion assays primarily reflect the diffusion of released ionic species rather than the migration of solid particles. Accordingly, the agar diffusion method was employed here as a qualitative tool to visualize antimicrobial effects and to complement the quantitative MIC results obtained by broth microdilution.

## 5 Cytotoxicity of the Nanozeolites

### 5.1 Cytotoxicity Assessment of Silver‐Exchanged Zeolite

The in vitro cytocompatibility of the silver‐exchange zeolite was evaluated using an extract‐based assay in accordance with established biomaterials testing principles and consistent with ISO 10993‐5 guidelines for biological evaluation of medical materials. An extraction protocol was employed to assess the potential cytotoxic effects of soluble species released from the material, including silver ions, under physiological‐like conditions. Zeolite extracts were prepared by incubating 1 mg of the material in 1 mL of Dulbecco's Modified Eagle Medium (DMEM), corresponding to an extraction ratio of 1 mg mL^−^^1^, at 37°C for 24 h under sterile conditions. Following incubation, suspensions were centrifuged to sediment particulate matter, and the supernatants were collected and filtered through a 0.22 µm membrane to ensure complete removal of residual solid particles. The resulting conditioned medium was considered the stock extract (1 mg mL^−^^1^) and was subsequently serially diluted in complete culture medium to obtain final test concentrations ranging from 1000 to 3.9 µg mL^−^^1^. Cell culture and viability assays were performed following the protocol described by Mena‐Silva et al. [42], with adaptations. Immortalized human keratinocytes (HaCaT cell line) [43], widely used as an epithelial model for skin‐contact biomaterials, were cultured in DMEM supplemented with 10% fetal bovine serum and 1% antibiotic/antifungal solution. Cells were maintained at 37°C in a humidified atmosphere containing 5% CO_2_. For viability testing, cells were seeded into 96‐well plates at a density of 7 × 10^3^ cells per well and allowed to adhere for 24 h to reach logarithmic growth phase. The culture medium was then replaced with medium containing the zeolite extracts at the specified concentrations, and cells were incubated for an additional 24 h. Cell metabolic activity was quantified using the MTS assay (CellTiter 96 AQueous One Solution Cell Proliferation Assay, Promega), which measures mitochondrial enzymatic reduction of the tetrazolium compound to a soluble formazan product. After 24 h exposure, 20 µL of MTS reagent was added to each well, and plates were incubated at 37°C for 1 h according to the manufacturer's instructions. Absorbance was measured at 450 nm using a Multiskan microplate reader. All experiments were performed in triplicate and repeated independently to ensure reproducibility. Cell viability was calculated as a percentage relative to the negative control (untreated cells), which was defined as 100% viability. According to ISO 10993‐5 criteria, materials are considered noncytotoxic when cell viability remains above 70% of the negative control. Statistical analysis was performed using one‐way analysis of variance (ANOVA) followed by Dunnett's post hoc test to compare treated groups with the control. Differences were considered statistically significant at *p* ≤ 0.05.

## 6 Results and Discussion

### 6.1 Structural Analysis by XRD

**FIGURE 1:**
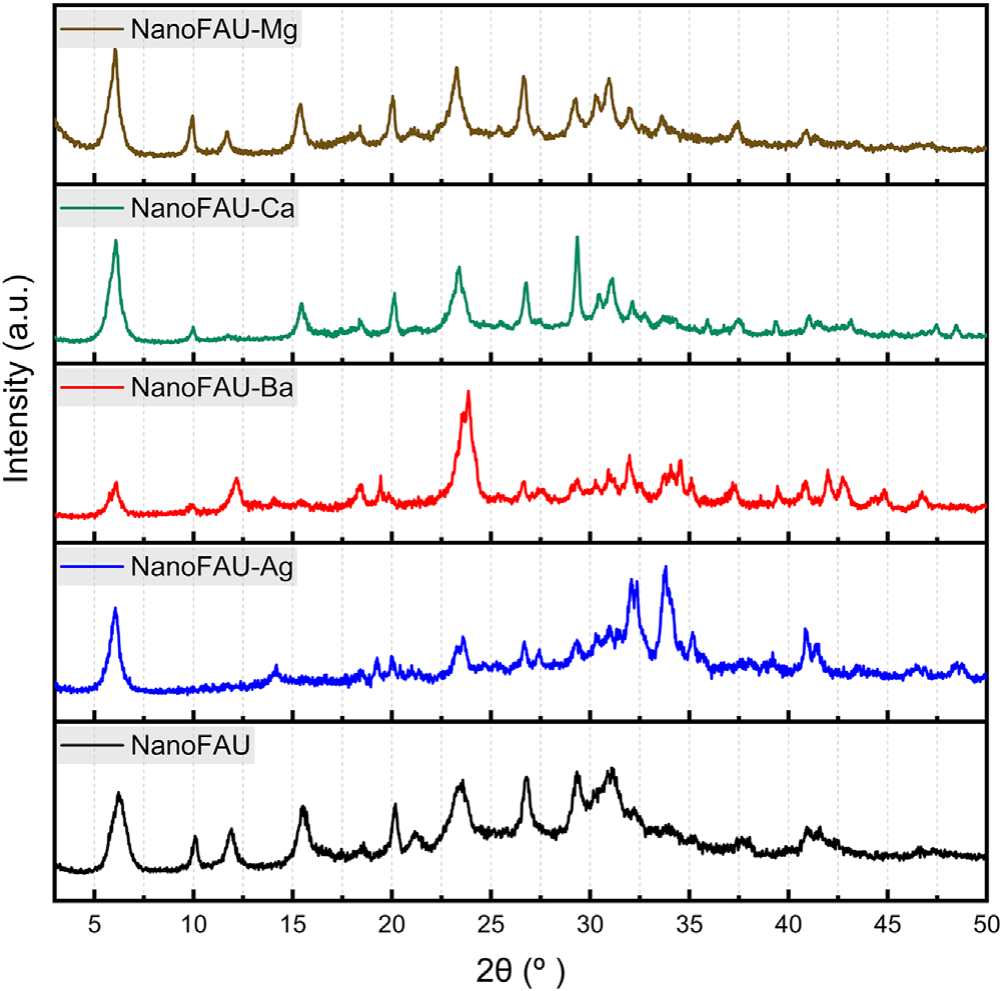
Powder XRD patterns of nanocrystalline Faujasite in the sodium form (NanoFAU) and its ion‐exchanged derivatives (Mg^2^^+^, Ca^2^^+^, Ag^+^, and Ba^2^^+^).

The structural properties of the synthesized materials were primarily evaluated by powder XRD. Figure 1 displays the diffraction patterns of the sodium form of NanoFAU and its ion‐exchanged derivatives. The pristine NanoFAU sample exhibits a diffraction pattern fully consistent with the FAU framework, in excellent agreement with reference data reported for nanocrystalline NaX‐type materials [31]. The characteristic Bragg reflections observed at 2*θ* ≈ 6.1°, 9.9°, 11.7°, 15.3°, 20.0°, 23.3°, 26.5°, and 31.1° confirm the successful formation of the FAU structure and indicate a high degree of crystallinity despite the nanometric crystal size.

Ion exchange with divalent magnesium (NanoFAU‐Mg) did not induce detectable changes in peak positions, indicating preservation of the FAU lattice parameters. The diffraction pattern remains essentially identical to that of the parent NanoFAU, apart from a moderate and systematic reduction in peak intensities. A similar behavior was observed for the calcium‐exchanged sample (NanoFAU‐Ca). In both cases, the attenuation of Bragg intensities can be attributed to increased x‐ray scattering disorder associated with partial replacement of Na^+^ by divalent cations, as well as subtle changes in electron density distribution within the supercages, rather than to framework collapse. These results indicate that exchange with alkaline‐earth cations of moderate ionic radius and hydration energy preserves the long‐range crystallographic order of the FAU framework.

In contrast, ion exchange with silver (NanoFAU‐Ag) resulted in pronounced modifications of the diffraction pattern. Several characteristic reflections, notably those at 2*θ* ≈ 9.8°, 11.6°, 15.3°, 20.0°, and 26.6°, are severely attenuated or no longer discernible, accompanied by a substantial decrease in the intensity of the remaining peaks. This behavior indicates a significant loss of long‐range crystalline order, consistent with partial amorphization or increased structural disorder at the nanocrystal scale. The disruption induced by Ag^+^ exchange is likely associated with the high polarizability, distinct coordination preferences, and strong framework–cation interactions of silver ions, which can locally distort the aluminosilicate lattice and destabilize periodic ordering.

A comparable, albeit less pronounced, effect was observed for the barium‐exchanged sample (NanoFAU‐Ba), which exhibits attenuation or loss of reflections at 2*θ* ≈ 9.8°, 15.3°, and 20.0°, together with a general reduction in diffraction intensity. Given the large ionic radius and lower hydration energy of Ba^2^^+^ relative to Ca^2^^+^ and Mg^2^^+^, these results suggest that steric effects and reduced mobility within the FAU supercages contribute to framework strain and partial loss of crystallographic coherence.

Overall, the XRD results demonstrate that the structural stability of NanoFAU upon ion exchange is strongly dependent on the nature of the compensating cation. While exchange with Mg^2^^+^ and Ca^2^^+^ preserves the FAU framework with only minor reductions in crystallinity, incorporation of Ag^+^ and Ba^2^^+^ induces significant disruption of long‐range order. Importantly, the persistence of residual FAU reflections in the latter samples indicates that the framework is not completely collapsed, but rather transformed into a more disordered or defect‐rich state, a feature that may have direct implications for ion release behavior and biofunctional performance discussed in subsequent sections.

### 6.2 Fourier‐transform infrared (FT‐IR) Analysis

FT‐IR spectroscopy was employed to examine the framework integrity of NanoFAU in its sodium form and after ion exchange with different cations. The FAU structure consists of interconnected TO_4_ tetrahedra (*T* = Si or Al), whose vibrational modes generate characteristic absorption bands in the mid‐ and far‐infrared regions. These vibrations are commonly classified as internal modes of the TO_4_ tetrahedra and external modes associated with framework linkages and ring structures [44].

The fundamental framework vibrations of aluminosilicate zeolites occur predominantly in the 1300–400 cm^−^^1^ region, which defines the structural fingerprint of the FAU topology [44, 45]. In this range, the most prominent bands correspond to asymmetric T–O–T stretching vibrations (typically 1250–950 cm^−^^1^), symmetric stretching modes (approximately 820–750 cm^−^^1^), and bending or linkage vibrations in the 650–420 cm^−^^1^ region. External vibrations associated with double six‐membered rings and pore‐opening structures are generally observed below 600 cm^−^^1^ [44, 45].

**FIGURE 2:**
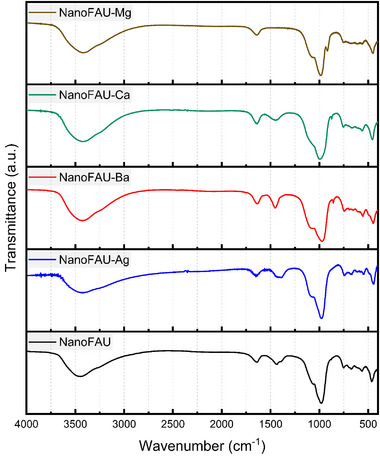
Fourier‐transform infrared (FT‐IR) spectra of nanocrystalline Faujasite in the sodium form (NanoFAU) and its ion‐exchanged derivatives (Mg^2^^+^, Ca^2^^+^, Ag^+^, and Ba^2^^+^).

As shown in Figure 2, the FT‐IR spectra of NanoFAU and its ion‐exchanged derivatives display well‐defined absorption bands at approximately 980, 750, 670, 560, and 460 cm^−^^1^. The band centered near 980 cm^−^^1^ is attributed to asymmetric stretching of T–O–T linkages, while features at 750–670 cm^−^^1^ arise from symmetric stretching modes of the tetrahedral units. The bands at ∼560 and ∼460 cm^−^^1^ are characteristic of double‐ring vibrations and T–O bending modes, respectively, confirming preservation of the FAU framework [44, 45].

Broad absorption bands observed at 1630–1650 and 3400–3500 cm^−^^1^ correspond to H–O–H bending and O–H stretching vibrations of physisorbed and intracrystalline water molecules confined within the zeolitic cavities. The persistence of these bands is consistent with the hydrophilic character of the aluminosilicate framework.

Importantly, ion exchange with Mg^2^^+^, Ca^2^^+^, Ag^+^, and Ba^2^^+^ did not induce significant shifts in the principal framework vibration frequencies within the accessible spectral range (400–4000 cm^−^^1^), indicating that the tetrahedral aluminosilicate skeleton remained largely intact after cation substitution. The low‐frequency region below 400 cm^−^^1^, where cation–framework interactions and extra‐framework metal–oxygen vibrations are typically observed, was not accessible under the present experimental conditions [44, 45]. Nonetheless, a systematic attenuation of band intensities was noted for certain exchanged samples, particularly NanoFAU‐Ag, suggesting increased structural disorder or modification of local electronic environments, in agreement with the XRD results.

All NanoFAU samples, including the sodium form and ion‐exchanged derivatives, exhibited an additional absorption feature in the 1400–1490 cm^−^^1^ region. This band is not commonly reported for microcrystalline FAU materials. A similar unassigned feature has been described for nanometric FAU‐type zeolites (NanoX) by Azizi et al. [46]. In the present case, this band may be tentatively attributed to surface‐related species, possibly associated with an increased density of silanol (Si─OH) and aluminol (Al─OH) groups resulting from the high surface‐to‐volume ratio characteristic of nanocrystalline zeolites. Alternatively, weak contributions from residual carbonate species adsorbed from atmospheric CO_2_ cannot be completely excluded. Further investigation would be required to definitively assign this feature.

### 6.3 Morphology and Elemental Composition

To investigate the morphology, particle size, and compositional characteristics of the synthesized materials, scanning and transmission electron microscopy (SEM/HRTEM), AFM, and EDS were employed in a complementary manner.

**FIGURE 3:**
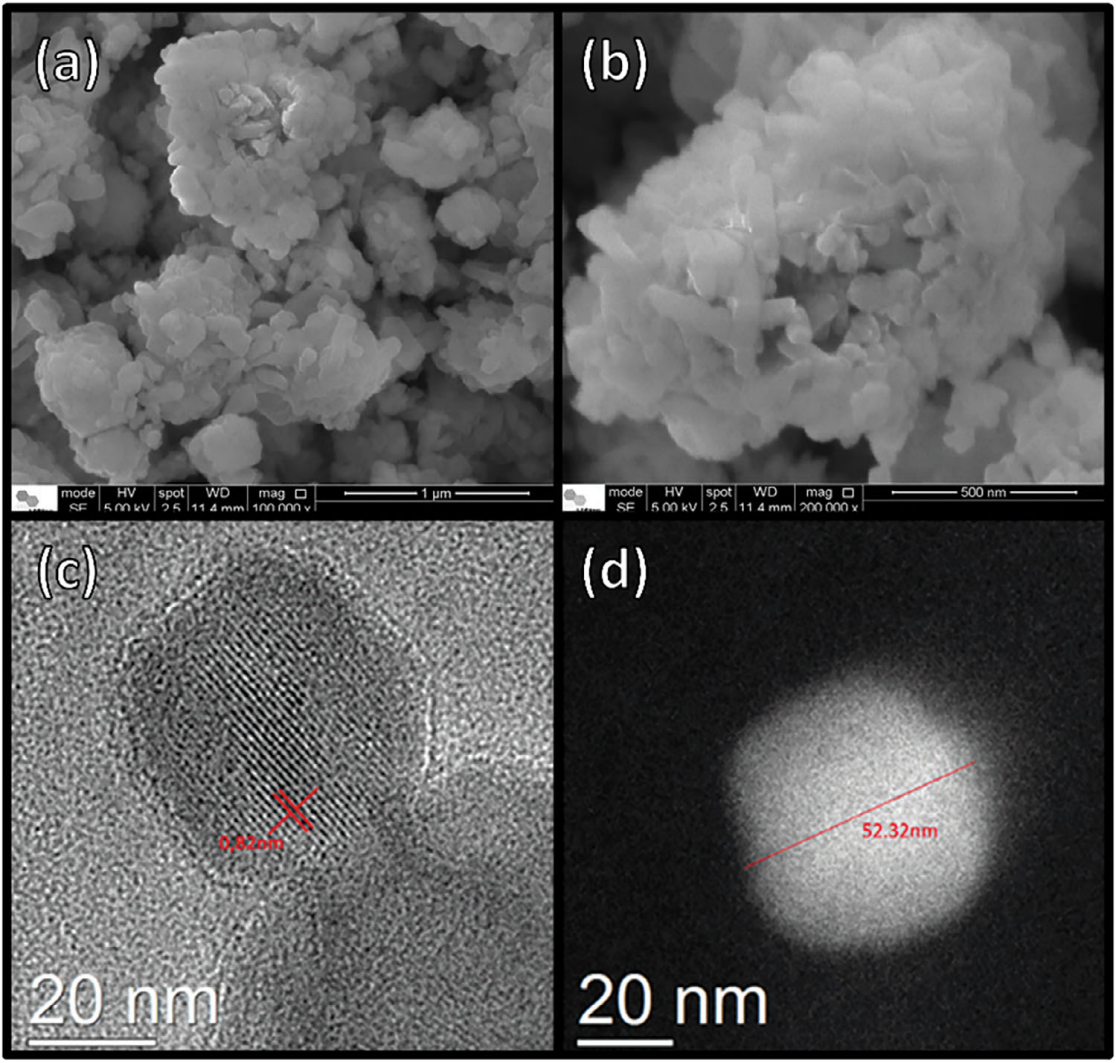
SEM (a, b) and HRTEM (c, d) images of nanocrystalline Faujasite (NanoFAU). SEM reveals aggregated assemblies composed of primary nanocrystals. HRTEM shows well‐resolved lattice fringes (*d* ≈ 0.82 nm) characteristic of the FAU framework, while dark‐field TEM indicates representative particle size of approximately 52 nm, corroborating the data reported by Zhan et al. [31] when first describing the synthetic route adopted in this work.

Representative SEM and HRTEM images of the as‐synthesized NanoFAU are presented in Figure 3. SEM analysis (Figure 3a–b) reveals that the material consists of secondary aggregates ranging from approximately 0.3 to 1 µm, formed by the assembly of primary nanocrystals. Higher‐magnification SEM images clearly show that the aggregate surfaces are composed of discrete nanosized domains with estimated diameters in the 30–80 nm range, confirming the nanometric nature of the synthesized zeolite.

High‐resolution TEM analysis (Figure 3c) further demonstrates well‐defined lattice fringes, indicating high crystallographic order at the nanoscale. The measured interplanar spacing of approximately 0.82 nm is consistent with characteristic lattice planes of FAU‐type zeolites, such as the (331) or (220) reflections, confirming preservation of the FAU framework after nanocrystal formation. The absence of amorphous regions supports the high structural integrity of the material. Furthermore, particle size analysis from TEM images (Figure 3d) shows individual crystals with diameters centered around ∼50 nm (representative particle: 52.32 nm), corroborating the data reported by Zhan et al. [31] when first describing the synthetic route adopted in this work.

Complementary AFM analysis (Figure S1) provides additional insight into the surface topography of the NanoFAU aggregates. The AFM height maps reveal densely packed nanocrystals with relatively smooth surface features at the micrometer scale. Although AFM can, in principle, resolve fine surface details, the extensive agglomeration observed in both SEM and AFM images limits the extraction of individual particle boundaries and precise surface roughness parameters. Nevertheless, the AFM data are fully consistent with the particle size and aggregation state inferred from electron microscopy.

**FIGURE 4:**
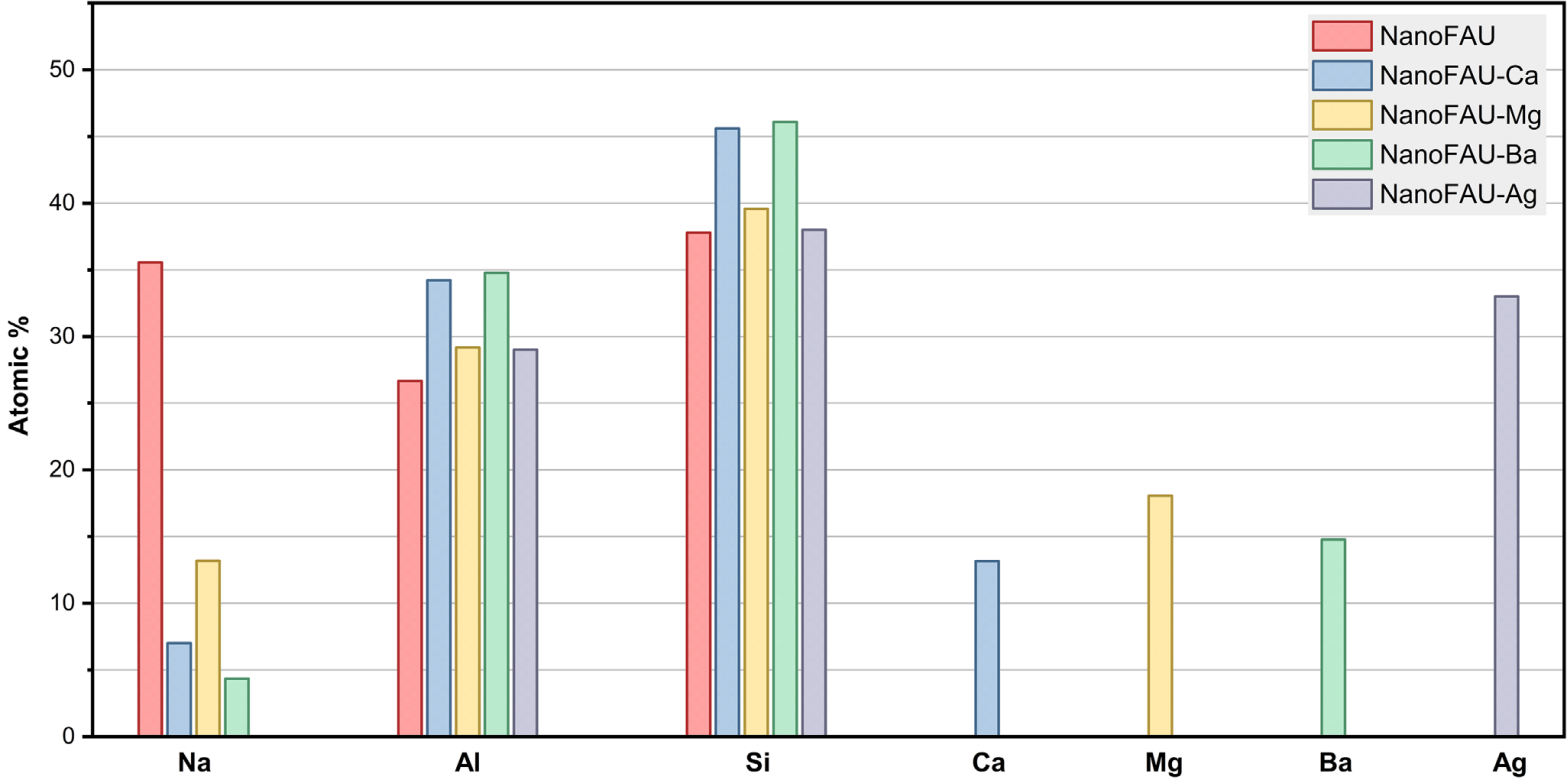
Elemental composition (atomic %) of nanocrystalline NaX and its ion‐exchanged derivatives, as determined by energy‐dispersive x‐ray spectroscopy (EDS) within the analyzed region.

Elemental composition and ion‐exchange efficiency were assessed by EDS. Figure 4 and Table 1 summarize the atomic percentages and normalized oxide compositions of NanoFAU and its ion‐exchanged derivatives. As expected, all samples exhibit aluminum and silicon as the principal framework‐forming elements, confirming the integrity of the aluminosilicate FAU backbone. Sodium is detected in the parent NanoFAU sample as the charge‐compensating extra‐framework cation, in agreement with the alkaline hydrothermal synthesis conditions employed.

Although EDS is inherently surface‐sensitive, compositional bias is not expected to significantly affect the present analysis for two reasons: (1) the high surface‐to‐volume ratio of the nanoparticles ensures that near‐surface composition is representative of the bulk, and (2) the electron beam penetration depth is comparable to the particle dimensions, enabling effective sampling of the entire nanocrystal volume. Consequently, EDS provides a reliable semi‐quantitative assessment of cation exchange in these materials.

To enable a chemically meaningful comparison of ion‐exchange efficiency among different samples, oxide compositions were normalized to Al_2_O_3_ (Table 1). This normalization is justified because the number of extra‐framework charge‐compensating cations is directly proportional to the number of framework [AlO_4_]^−^ units. The normalized compositions clearly reveal a systematic decrease in Na_2_O content accompanied by the incorporation of the corresponding exchange cations (Ca^2^^+^, Mg^2^^+^, Ba^2^^+^, or Ag^+^), confirming the success of the ion‐exchange process.

Among the exchanged samples, NanoFAU‐Ag exhibits complete replacement of Na^+^ by Ag^+^, corresponding to 100% exchange efficiency within experimental uncertainty. This behavior is consistent with the well‐documented high selectivity of Ag^+^ toward zeolitic frameworks, attributed to its strong polarizing power and high affinity for negatively charged framework sites [47, 48]. In contrast, partial exchange is observed for divalent cations, with exchange efficiencies of approximately 84.6% for Ca^2^^+^, 61.5% for Mg^2^^+^, and 92.3% for Ba^2^^+^. These differences reflect the combined influence of ionic radius, hydration energy, and mobility within the FAU supercages.

Notably, the apparent deviation from ideal charge compensation observed for NanoFAU‐Mg suggests a more complex distribution of Mg^2^^+^ species, potentially involving partial hydration or heterogeneous site occupancy. This behavior will be further discussed in the context of the zeta potential and IEP measurements, where surface charge effects become particularly relevant.

### 6.4 ^2^ ^9^Si MAS NMR and Framework Integrity

**FIGURE 5:**
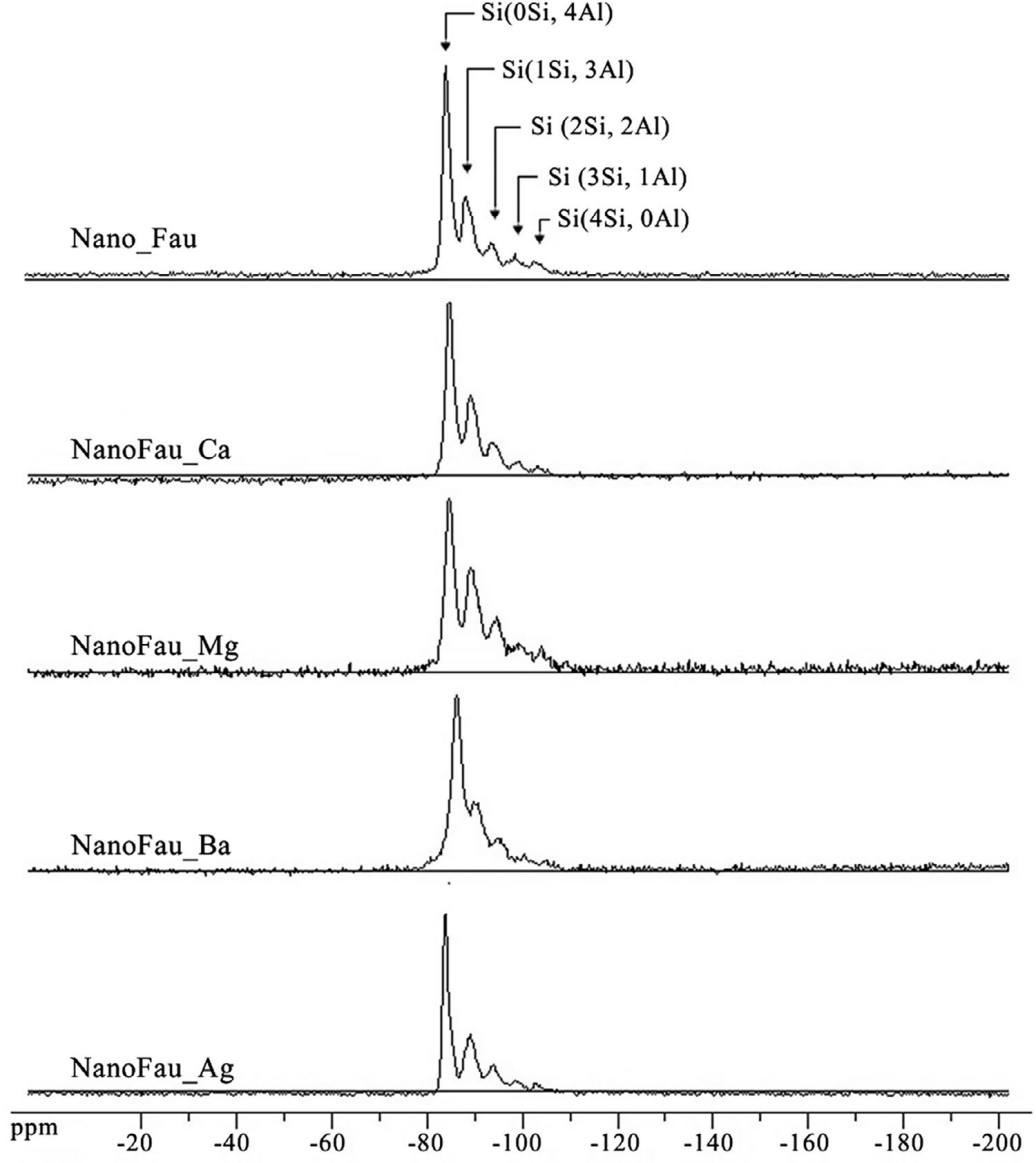
Solid‐state ^2^ ^9^Si magic‐angle spinning (MAS) NMR spectra of nanocrystalline Faujasite (NanoFAU) in the sodium form and its ion‐exchanged derivatives (Ca^2^^+^, Mg^2^^+^, Ba^2^^+^, and Ag^+^).

Figure 5 presents the solid‐state ^2^ ^9^Si magic‐angle spinning (MAS) NMR spectra of NanoFAU in its sodium form and after ion exchange with Ca^2^^+^, Mg^2^^+^, Ag^+^, and Ba^2^^+^. In all samples, the spectra are dominated by multiple resonances characteristic of silicon atoms in different local aluminosilicate environments within the FAU framework. The observed chemical shifts are in excellent agreement with those reported for FAU‐type zeolites in the literature [49, 50].

For the parent NanoFAU sample, five distinct resonances are clearly resolved. The signal centered at approximately −84 ppm is assigned to Si atoms coordinated to four aluminum neighbors through oxygen bridges, denoted as Si(0Si,4Al). Peaks at −89, −94, −99, and −103 ppm correspond to Si(1Si,3Al), Si(2Si,2Al), Si(3Si,1Al), and fully siliceous Si(4Si,0Al) environments, respectively. The presence of this full distribution of Q^4^(nAl) species confirms the characteristic aluminum ordering of the FAU framework and indicates the absence of framework dealumination during synthesis.

Upon ion exchange, the overall spectral features remain largely preserved for NanoFAU‐Ca, NanoFAU‐Mg, and NanoFAU‐Ag. No significant changes in the number of resonances or their relative positions are observed, indicating that substitution of Na^+^ by these cations does not perturb the tetrahedral coordination of silicon or the long‐range framework connectivity. This observation corroborates the XRD and FT‐IR results, collectively demonstrating that ion exchange proceeds without disruption of the aluminosilicate skeleton.

A slight but systematic downfield shift (approximately 1–2 ppm) is observed for NanoFAU‐Ba, with resonances appearing at −86, −90, −95, −100, and −104 ppm. These subtle changes are attributed to the influence of Ba^2^^+^ cations on the local electronic environment of the framework oxygen atoms, which indirectly affects the shielding of neighboring silicon nuclei. Given the large ionic radius and lower hydration energy of Ba^2^^+^ relative to Ca^2^^+^ and Mg^2^^+^, stronger electrostatic interactions with framework oxygen atoms are expected. Importantly, the magnitude of these shifts is small and does not indicate framework rearrangement, dealumination, or changes in silicon coordination.

Overall, the ^2^ ^9^Si MAS NMR results unequivocally demonstrate that the FAU framework remains structurally intact after ion exchange with all investigated cations. The persistence of well‐resolved Q^4^(nAl) environments confirms that cation substitution primarily affects extra‐framework sites, while the tetrahedral aluminosilicate network is preserved. These findings provide a critical structural basis for interpreting subsequent variations in surface charge, textural properties, and biological performance.

### 6.5 Textural Properties, Hierarchical Porosity, and Surface Charge

Nitrogen adsorption–desorption isotherms of NanoFAU (Figure S2) exhibit a Type IV profile according to IUPAC classification, characterized by a distinct hysteresis loop at intermediate‐to‐high relative pressures (*P*/*P*_0_). This behavior indicates the presence of mesoporosity in addition to the intrinsic microporosity of the FAU framework. The hysteresis loop is consistent with interparticle mesopores formed by aggregation of nanocrystals, as observed in SEM and AFM analyses.

Although classical Type I uptake associated with purely microporous zeolites is not sharply resolved, this does not indicate loss of intrinsic FAU microporosity. Rather, the dominant mesoporous contribution reflects the nanocrystalline nature of the material and the formation of intercrystallite voids. The preservation of the aluminosilicate framework microporosity is independently confirmed by XRD and ^2^ ^9^Si MAS NMR, which demonstrate structural integrity and absence of framework collapse or dealumination.

BJH pore size distribution analysis (Figure S3) reveals a broad distribution with a significant contribution in the 20–50 nm range, attributable to interparticle mesopores. A pronounced feature centered at an apparent pore radius of ∼1.7 nm is also observed. This contribution arises from the convolution of framework‐defined micropores with narrow interparticle voids, characteristic of aggregated nanocrystals. The resulting architecture can therefore be described as hierarchical micro–mesoporous.

From a functional perspective, such hierarchical porosity is advantageous for blood‐contacting applications. The combination of intrinsic micropores and mesoscopic interparticle voids facilitates rapid fluid uptake, high accessible surface area, and enhanced adsorption of plasma proteins and clotting factors, while maintaining the capacity for controlled release of exchangeable cations.

### 6.6 Surface Charge and Isoelectric Behavior

**FIGURE 6:**
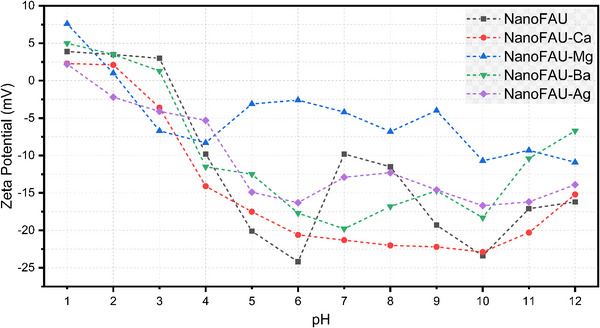
Zeta potential profiles of nanocrystalline Faujasite in the sodium form (NanoFAU) and its ion‐exchanged derivatives (Ca^2^^+^, Mg^2^^+^, Ba^2^^+^, and Ag^+^) as a function of pH, measured in CaCl_2_ solution, highlighting their respective isoelectric points.

Surface charge density critically governs the interaction between inorganic materials and biological systems, particularly in the context of blood coagulation. The electrostatic behavior of the materials was evaluated through zeta potential measurements as a function of pH in CaCl_2_ solution (Figure 6), approximating physiological ionic conditions.

In zeolitic systems, surface charge arises from protonation–deprotonation equilibria of surface silanol and aluminol groups. At pH values above the IEP, deprotonation leads to negatively charged SiO^−^ and AlO^−^ sites, whereas at pH values below the IEP the surface becomes neutral or positively charged [34, 51]. The relative position of the IEP with respect to physiological blood pH (∼7.4) is particularly relevant for predicting hemostatic behavior [33, 52].

All ion‐exchanged samples exhibit a downward shift of the IEP relative to the parent NanoFAU (IEP ≈ 3.2). Specifically, the IEP values are approximately 2.5 (NanoFAU‐Ca), 2.3 (NanoFAU‐Mg), 3.1 (NanoFAU‐Ba), and 1.5 (NanoFAU‐Ag). Consequently, under physiological conditions all materials possess a net negative surface charge, favoring electrostatic interactions with positively charged domains of coagulation factors.

It is worth noting that NanoFAU‐Mg displays relatively more positive zeta potential values across a broad pH range. This behavior is consistent with its nonideal charge compensation inferred from normalized oxide composition (1.0 Al_2_O_3_:2.7 SiO_2_:0.5 Na_2_O:1.2 MgO), which implies an effective excess of extra‐framework cationic charge. This excess likely contributes to partial surface charge neutralization and modifies the electrokinetic profile.

Collectively, these results demonstrate that ion exchange not only preserves the structural integrity of the FAU framework but also modulates both hierarchical porosity and surface charge characteristics. These physicochemical modifications provide the mechanistic foundation for the distinct hemostatic and antimicrobial behaviors observed among the NanoFAU derivatives.

### 6.7 Thromboelastographic Evaluation

**FIGURE 7:**
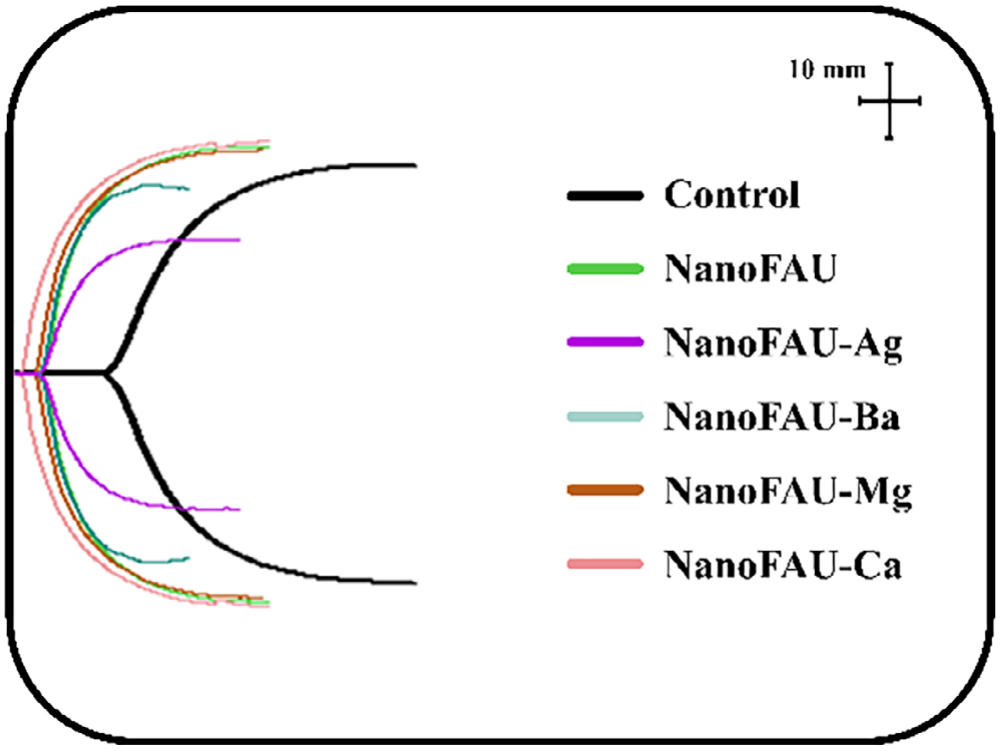
Thromboelastography (TEG) curves of citrated whole blood before zeolite addition (control) and after treatment with nanocrystalline Faujasite (NanoFAU) and its ion‐exchanged derivatives (NanoFAU‐Ca, NanoFAU‐Mg, NanoFAU‐Ba, and NanoFAU‐Ag).

The hemostatic performance of NanoFAU and its ion‐exchanged derivatives was systematically evaluated by TEG, a technique that provides real‐time, quantitative assessment of the viscoelastic evolution of clot formation in whole blood. Citrated whole blood without zeolite addition was used as the control, enabling direct comparison with samples treated with NanoFAU, NanoFAU‐Ca, NanoFAU‐Mg, NanoFAU‐Ba, and NanoFAU‐Ag. Representative TEG tracings are shown in Figure 7, and the corresponding quantitative parameters are summarized in Table 2.

**TABLE 2:** Thromboelastographic (TEG) parameters of citrated whole blood in the absence of zeolite (control) and after treatment with nanocrystalline Faujasite (NanoFAU) and its ion‐exchanged derivatives (NanoFAU‐Ca, NanoFAU‐Mg, NanoFAU‐Ba, and NanoFAU‐Ag). Reaction time (*R*), clot formation time (*K*), *α*‐angle, and maximum amplitude (MA) were extracted from TEG tracings and are reported as mean ± standard deviation (*n* = 3).

	*R* (min)	*K* (min)	Alpha Angle (°)	MA (mm)
Reference value	5–10	1–3	45–74	54–62
Control	8.6 ± 0.7	3.5 ± 0.6	49.7 ± 6.8	53.6 ± 2.4
NanoFAU	2.4 ± 0.1	1.2 ± 0.0	73.9 ± 0.5	61.2 ± 1.6
NanoFAU‐Ca	1.1 ± 0.2	1.2 ± 0.1	75.1 ± 2.3	60.3 ± 1.6
NanoFAU‐Mg	2.1 ± 0.2	1.1 ± 0.2	76.5 ± 2.2	53.5 ± 5.7
NanoFAU‐Ba	2.7 ± 0.2	1.0 ± 0.1	75.6 ± 0.3	50.9 ± 3.4
NanoFAU‐Ag	3.4 ± 0.5	2.5 ± 0.6	60.0 ± 2.4	42.4 ± 6.4

The analyzed TEG parameters included the reaction time (*R*), reflecting the latency to initial fibrin formation; the clot formation time (*K*), associated with clot propagation kinetics; the *α*‐angle, indicative of the rate of fibrin network growth; and the MA, which reflects the final clot strength arising from fibrin–platelet interactions.

Relative to the control, all zeolitic materials significantly accelerated the initiation of coagulation, as evidenced by a pronounced reduction in R values. Pristine NanoFAU reduced the R time from 8.6 ± 0.7 min (control) to 2.4 ± 0.1 min, confirming its intrinsic procoagulant activity. This effect was further modulated by ion exchange. Among the derivatives, NanoFAU‐Ca exhibited the most pronounced acceleration of clot initiation, with an *R* value of 1.1 ± 0.2 min, approaching the lower limit of the physiological reference range and well below the threshold considered critical for rapid hemorrhage control.

NanoFAU‐Mg also demonstrated enhanced coagulation kinetics, with reduced *R* (2.1 ± 0.2 min) and *K* (1.1 ± 0.2 min) values and an increased *α*‐angle (76.5 ± 2.2°), indicating efficient fibrin build‐up. However, a modest reduction in MA (53.5 ± 5.7 mm) suggests that, although clot initiation and propagation were accelerated, the final clot strength was not proportionally enhanced. A similar trend was observed for NanoFAU‐Ba, which displayed slightly prolonged R values (2.7 ± 0.2 min) relative to NanoFAU‐Ca and NanoFAU‐Mg, combined with reduced MA (50.9 ± 3.4 mm), indicating weaker clot stabilization.

In contrast, NanoFAU‐Ag induced the most substantial impairment of clot formation. This sample exhibited prolonged *R* (3.4 ± 0.5 min) and *K* (2.5 ± 0.6 min) times, accompanied by a marked decrease in *α*‐angle (60.0° ± 2.4°) and a pronounced reduction in MA (42.4 ± 6.4 mm). The significant decrease in MA indicates compromised clot mechanical integrity, likely arising from disrupted fibrin polymerization and weakened platelet–fibrin interactions. These findings suggest that, although silver ion exchange confers strong antimicrobial functionality, it negatively interferes with the coagulation cascade.

Statistical analysis by one‐way ANOVA revealed highly significant differences in *R* values among all treated samples relative to the control (*p* < 0.0001), with NanoFAU‐Ca exhibiting the most pronounced effect. Significant differences were also observed for *K* and *α*‐angle (*p* < 0.005). In contrast, despite clear numerical trends, MA differences did not reach statistical significance, reflecting the larger variability inherent to clot strength measurements.

Importantly, the thromboelastographic findings were corroborated by phase‐contrast microscopy, which provided direct qualitative evidence of clot formation at the cellular level. As shown in Figures S4–S6, whole blood exposed to NanoFAU and its ion‐exchanged derivatives exhibited pronounced red blood cell aggregation and the formation of fibrin‐rich clot structures, in clear contrast to the control. These microstructural observations confirm that the accelerated coagulation kinetics detected by TEG arise from genuine fibrin network formation and cellular organization, rather than from purely physicochemical alterations of blood viscoelasticity.

The strong clot‐accelerating behavior observed here is consistent with the growing body of evidence that inorganic surfaces can be “functionally procoagulant” when engineered for rapid, localized contact activation and factor enrichment at the wound interface. Recent reviews emphasize that next‐generation hemostats increasingly leverage high surface area, tailored surface charge, and hierarchical porosity—features shared by nanozeolites, mesoporous silicas, and certain clay/mineral platforms—to concentrate proteins and promote intrinsic pathway activation without relying on biological coagulants [14, 15, 51, 52, 53]. It is already well established that polar inorganic surfaces, such as oxides and silicate‐based materials, can accelerate blood coagulation via the so‐called “glass effect” [33, 34, 51]. In this context, negatively charged surfaces act as contact activators for coagulation factor XII (FXII), initiating the intrinsic pathway. FXII activation occurs through electrostatic interactions between negatively charged surfaces and positively charged residues on the heavy chain of the zymogen, inducing conformational changes that promote autoactivation and subsequent proteolytic cleavage [36, 37, 54].

Beyond the traditional description of “negatively charged surface + Ca^2^^+^ supplementation”, protein adsorption (and subsequent corona formation) and surface‐mediated assembly of coagulation complexes are now also recognized as decisive contributors to the hemostatic response of calcium‐containing inorganic materials [14, 52]. Recently, Shang et al. [15] reported that Ca‐exchanged Y zeolite surfaces can “mimic” certain platelet functions by serving as platforms for prothrombinase complex assembly, effectively acting as “inorganic platelets.” Notably, the zeolite‐assembled prothrombinase complexes displayed enhanced activity, and the presence of Ca^2^^+^ was essential for efficient complex formation, consistent with its established physiological role in coagulation. Further adding to the complexity of these systems, several studies have shown that metal ions can directly modulate coagulation protein interactions and contact activation [55]. Mutch, Waters, and Morrissey [38], for instance, demonstrated that divalent metal ions immobilized on surfaces strongly interact with FXI, FXII, and high‐molecular‐weight kininogen, affecting activation of the contact pathway.

These concepts align with our findings that ion identity (e.g., Ca^2^^+^ vs. Ag^+^) can enhance early clotting kinetics while also modulating clot mechanics, highlighting the need to treat “procoagulant performance” as a coupled outcome of porosity‐driven factor concentration and ion/surface‐regulated protein interactions [14, 15, 52].

Taken together, the combined thromboelastographic and microscopic evidence demonstrates that NanoFAU exhibits strong intrinsic hemostatic activity, which can be further tuned by ion exchange. Among the tested materials, NanoFAU‐Ca emerged as the most promising candidate for rapid hemorrhage control, combining accelerated clot initiation with preserved clot strength. Conversely, NanoFAU‐Ag, while advantageous for antimicrobial purposes, significantly compromises coagulation performance, underscoring the need to balance hemostatic and antimicrobial functionalities in the rational design of multifunctional wound‐care materials.

It is important to distinguish intentional, localized procoagulant activity designed for topical hemostasis from broader systemic hemocompatibility requirements. According to ISO 10993‐4, comprehensive hemocompatibility evaluation of blood‐contacting materials includes assessment of hemolysis, platelet activation, coagulation pathway perturbation, and complement activation, particularly for devices intended for prolonged intravascular exposure [56]. In the present study, the materials are intended for topical application, where controlled surface‐mediated coagulation at the wound interface represents a desired therapeutic outcome rather than an indicator of pathological thrombogenicity. While dedicated hemolysis and platelet activation assays represent important next steps toward full translational validation, the absence of abnormal thromboelastographic signatures and the preservation of physiologically relevant clot strength parameters indicate that the materials do not induce uncontrolled coagulation under the investigated conditions.

Another practical motivation for nanoscale inorganic hemostats is mitigation of heat‐related tissue injury historically associated with strongly exothermic water adsorption in dehydrated zeolite powders. While direct temperature‐rise measurements were outside the scope of this work, prior calorimetric evidence shows that ion exchange can substantially reduce hydration enthalpy in zeolitic systems, supporting a materials‐design pathway to lower heat release during use [16, 57]. More broadly, the field is converging on porous inorganic architectures (including mesoporous silica microspheres) that combine rapid fluid uptake with improved thermal handling, reinforcing the translational relevance of nano‐/mesoporous designs for emergency hemostasis [53].

### 6.8 Antimicrobial Activity and Cytocompatibility

The antimicrobial performance of NanoFAU and its ion‐exchanged derivatives was systematically evaluated against *S. aureus* and *C. albicans* using broth microdilution (MIC), agar diffusion assays, and post‐incubation subculturing to discriminate between bacteriostatic/fungistatic and bactericidal effects.

Unmodified NanoFAU exhibited no detectable antimicrobial activity against either *S. aureus* or *C. albicans* at concentrations up to 0.1 g (Table 3), confirming that the sodium form of the zeolite framework is biologically inert with respect to microbial inhibition. Similarly, NanoFAU‐Mg showed no inhibitory effect at any tested concentration, indicating that magnesium incorporation does not confer antimicrobial functionality under the present experimental conditions.

**TABLE 3:** Antimicrobial activity of NanoFAU and its ion‐exchanged derivatives (NanoFAU‐Ag, NanoFAU‐Cu, and NanoFAU‐Mg) at concentrations of 0.1, 0.01, and 0.001 g against *Staphylococcus aureus* and *Candida albicans*.

	Inhibitory concentration (g)	
Material	*S. aureus*	*C. albicans*
NanoFAU	NI	NI
NanoFAU‐Ag	0.1	0.1
	0.01	0.01
	0.001	0.001
NanoFAU‐Mg	NI	NI

**Abbreviation**: NI, no inhibition.

In contrast, silver‐exchanged NanoFAU (NanoFAU‐Ag) displayed pronounced antimicrobial activity against both microorganisms. In the microdilution assay (Table 4), NanoFAU‐Ag inhibited *S. aureus* growth at concentrations as low as 1.0 and 0.5 mg mL^−^^1^, representing a reduction of more than two orders of magnitude relative to the concentrations required for growth inhibition by unmodified NanoFAU (250–500 mg mL^−^^1^). Subculturing aliquots from inhibited wells onto Mueller–Hinton agar yielded no colony growth, demonstrating that the antibacterial effect of NanoFAU‐Ag against *S. aureus* is bactericidal.

**TABLE 4:** Inhibitory concentrations of zeolites against *S. aureus* and *C. albicans* observed in the microdilution antimicrobial susceptibility test.

	Inhibitory concentration (mg/mL)	
Material	*S. aureus*	*C. albicans*
NanoFAU	500	NI
	250	NI
NanoFAU‐Ag	1	1
	0.5	0.5

**Abbreviation**: NI, no inhibition.

For *C. albicans*, NanoFAU‐Ag also inhibited growth at 1.0 and 0.5 mg mL^−^^1^ in the microdilution assay. However, subsequent subculturing revealed colony formation at all concentrations, including those initially showing inhibition. This behavior indicates a fungistatic effect, whereby fungal proliferation is suppressed only during direct contact with the silver‐exchanged zeolite. The absence of irreversible growth inhibition highlights a pathogen‐dependent response to silver ion release.

**FIGURE 8:**
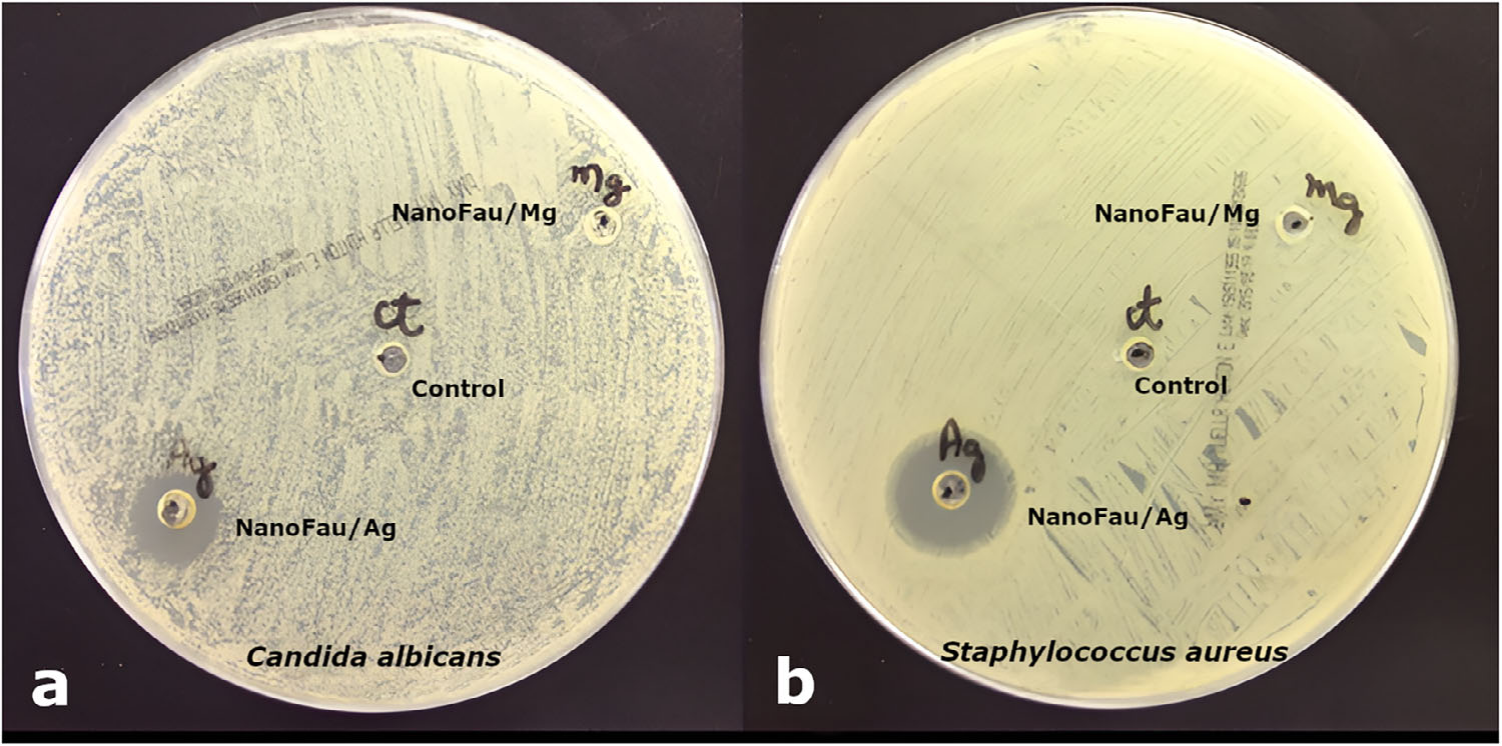
Representative inhibition halos observed in agar diffusion assays using nanometric zeolite suspensions (4 mg mL^−^^1^). (a) Antifungal activity against *Candida albicans*: NanoFAU‐Zn (no inhibition), NanoFAU‐Mg (no inhibition), NanoFAU‐Ag (inhibition halo: 19.8 mm), NanoFAU‐Cu (no inhibition), and growth control (CT). (b) Antibacterial activity against *Staphylococcus aureus*: NanoFAU‐Zn (no inhibition), NanoFAU‐Mg (no inhibition), NanoFAU‐Ag (inhibition halo: 25 mm), NanoFAU‐Cu (no inhibition), and growth control (CT).

**FIGURE 9:**
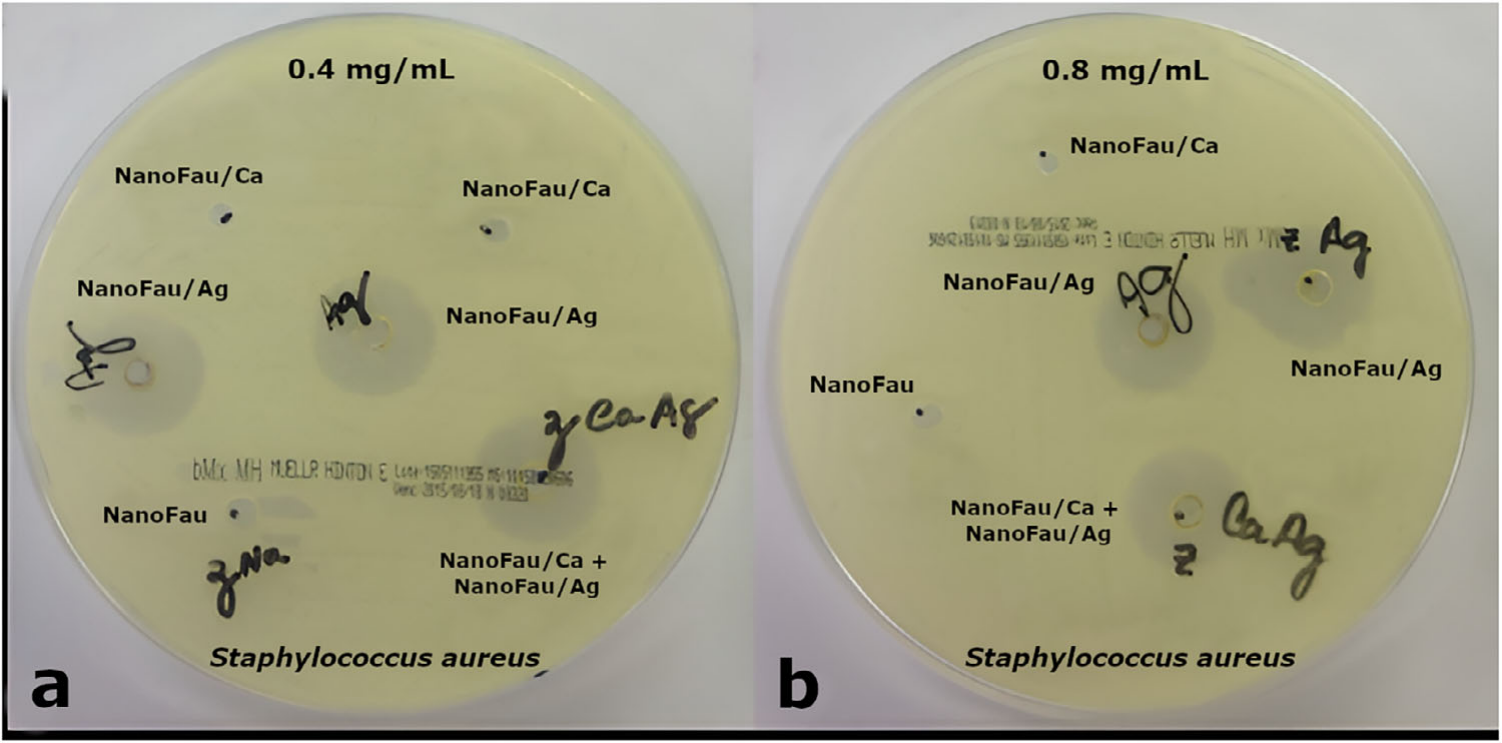
Antibacterial activity of zeolite‐based materials against *Staphylococcus aureus* evaluated by the agar diffusion method. (a) At 0.4 mg mL^−^^1^: NanoFAU (no inhibition), NanoFAU‐Ca (no inhibition), NanoFAU‐Ag (inhibition halo: 23 mm), and NanoFAU‐Ca + NanoFAU‐Ag (inhibition halo: 24 mm). (b) At 0.8 mg mL^−^^1^: NanoFAU (no inhibition), NanoFAU‐Ca (no inhibition), NanoFAU‐Ag (inhibition halo: 22 mm), and NanoFAU‐Ca + NanoFAU‐Ag (inhibition halo: 22 mm).

The antimicrobial activity was further corroborated by agar diffusion assays (Figures 8 and 9). NanoFAU‐Ag was the only material that produced clear inhibition halos against both microorganisms. Against *C. albicans*, NanoFAU‐Ag generated a halo of 19.8 mm (Figure 8a), whereas all other materials—including NanoFAU, NanoFAU‐Mg, NanoFAU‐Zn, and NanoFAU‐Cu—showed no detectable inhibition. For *S. aureus*, NanoFAU‐Ag produced a pronounced inhibition zone of approximately 25 mm (Figure 9b). Additional assays conducted at two zeolite concentrations (0.4 and 0.8 mg mL^−^^1^) confirmed that NanoFAU‐Ag consistently inhibited *S. aureus*, with inhibition halos ranging from 22 to 23 mm (Figure 9). A physical mixture of NanoFAU‐Ca and NanoFAU‐Ag exhibited inhibition halos comparable to NanoFAU‐Ag alone, whereas NanoFAU‐Ca by itself showed no antibacterial effect. These results demonstrate that the antimicrobial activity of the composite formulation arises exclusively from the silver‐exchanged component and is largely independent of the zeolite concentration within the investigated range.

The observed antimicrobial activity of NanoFAU‐Ag can be directly attributed to the presence of silver ions within the FAU framework. Zeolites act as reservoirs for Ag^+^ ions, which can be gradually released via ion exchange with competing cations present in biological media [18, 58, 59, 60]. Once released, silver ions exert antimicrobial effects through multiple, nonspecific mechanisms, including disruption of membrane integrity, inactivation of essential thiol‐containing enzymes, interference with nucleic‐acid replication, and induction of oxidative stress via reactive oxygen species generation [17, 60, 61, 62, 63].

Beyond simple ion release, the antimicrobial efficacy of NanoFAU‐Ag reflects the zeolite acting as a release‐modulating matrix under ionic competition, enabling sustained local Ag^+^ availability rather than an uncontrolled burst dose. Contemporary mechanistic studies emphasize that Ag‐zeolite activity arises from the interplay between (1) Ag^+^ release kinetics governed by ion‐exchange equilibria and hydration behavior, and (2) multi‐target microbial damage pathways, including membrane disruption, enzyme inactivation, nucleic‐acid interference, and oxidative stress processes [18, 26, 27, 58]. Importantly, recent work also supports a role for ROS‐associated oxidative activity in Ag‐exchanged zeolites under aerobic conditions, with measurable coupling between oxidative processes and antimicrobial efficacy that depends on zeolite structure, Ag loading, and environmental conditions [58]. In parallel, antibiofilm activity has been documented for Ag‐ion‐exchanged zeolites in contemporary microbiological models, reinforcing their relevance for wound‐infection control, where biofilm formation represents a major clinical barrier [18, 58, 59, 61, 62, 64].

The pathogen‐dependent response observed in this study—bactericidal against *S. aureus* and fungistatic against *C. albicans*—is consistent with previous reports indicating that microbial susceptibility to silver‐based materials is influenced by differences in cell wall architecture, metabolic activity, and oxidative stress tolerance [58, 59]. The robust antimicrobial response of NanoFAU‐Ag, combined with the absence of activity in sodium‐ and magnesium‐exchanged zeolites, confirms that silver incorporation is both necessary and sufficient to impart antimicrobial functionality to NanoFAU.

### 6.9 Cytocompatibility Assessment

**FIGURE 10:**
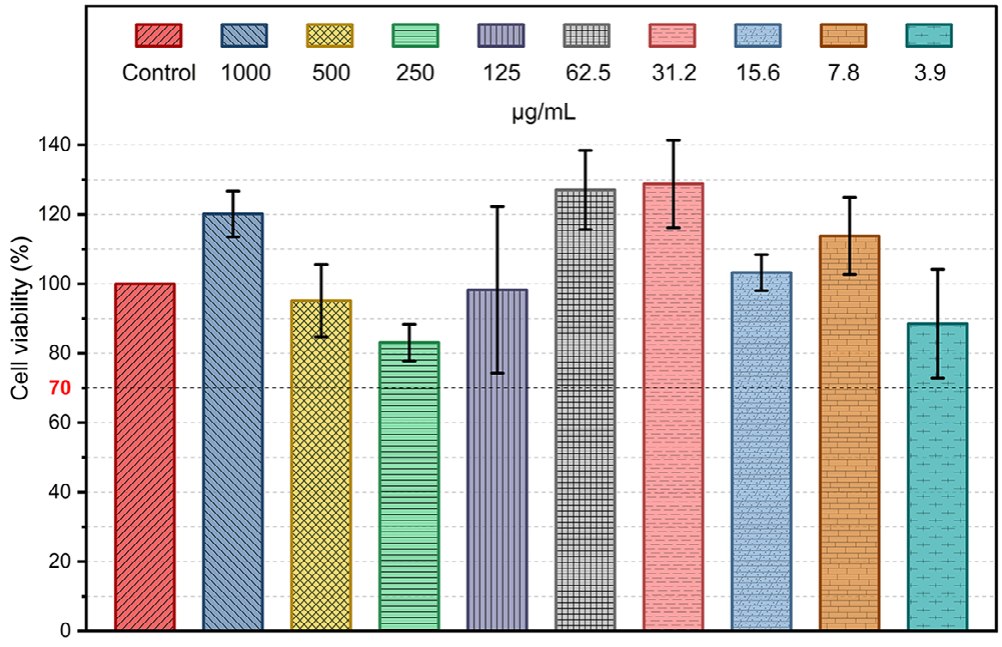
Cell viability (%) of HaCaT cells after 24 h exposure to silver‐exchanged zeolite at concentrations ranging from 1000 to 3.9 µg/mL. Data represent mean ± SD (*n* = 3). Statistical analysis was performed using one‐way ANOVA followed by Dunnett's post hoc test. ns: not significant (*p* > 0.05). The dashed line represents the 70% viability threshold according to ISO 10993‐5.

The cytotoxicity of NanoFAU‐Ag was evaluated in human keratinocyte (HaCaT) cells after 24 h exposure using the MTS assay. As shown in Figure 10, cell viability remained above 70% across all tested concentrations (1000 to 3.9 µg mL^−^^1^), with no statistically significant differences relative to the negative control (one‐way ANOVA followed by Dunnett's post hoc test, *p* > 0.05).

According to ISO 10993‐5, materials that reduce cell viability below 70% are classified as cytotoxic. Under the conditions investigated here, NanoFAU‐Ag did not meet this criterion, indicating a favorable in vitro biocompatibility profile toward epithelial cells. Given that HaCaT keratinocytes are a well‐established model for human epidermal tissue, these results support the suitability of silver‐exchanged FAU for applications involving direct contact with skin or wound environments.

## 7 Conclusions

NanoFAU and its ion‐exchanged derivatives were demonstrated to constitute a tunable inorganic biomaterial platform capable of integrating hemostatic and antimicrobial functionalities within a single framework. Structural characterization confirmed preservation of the FAU lattice upon ion exchange, while surface charge analysis revealed physiologically relevant negative surface potentials consistent with contact‐activation‐mediated coagulation.

Thromboelastographic evaluation identified calcium exchange as the most effective strategy for enhancing clot initiation and propagation, with NanoFAU‐Ca exhibiting rapid clot formation (*R* = 1.1 min) and preserved clot strength (MA = 60.3 mm). In contrast, silver exchange conferred potent antimicrobial activity, producing bactericidal effects against *S. aureus* and fungistatic inhibition of *C. albicans*, while maintaining cytocompatibility above ISO 10993‐5 thresholds. These findings highlight a cation‐dependent trade‐off between maximal coagulation efficiency and antimicrobial potency, underscoring the importance of ion identity in governing multifunctional performance. Overall, ion exchange emerges as a straightforward and effective strategy for rationally tailoring the biological behavior of NanoFAU. By decoupling and selectively optimizing coagulation and antimicrobial responses, NanoFAU derivatives represent promising candidates for advanced topical hemostats requiring simultaneous hemorrhage control and infection mitigation. Future studies focusing on in vivo validation and controlled ion‐release kinetics will be critical for translational development.

## Author Contributions

Guilherme de Paula Guarnieri and Juliana Bergamasco Laurenti were responsible for the synthesis and physicochemical characterization of the materials, data acquisition, data interpretation, and preparation of figures. Edivandra Buzato Silva, Beatriz Gonçalves Oliveira Crespo, and Taiza Maschio‐Lima performed the antimicrobial activity assays, including data acquisition and figure preparation. Eny Maria Goloni‐Bertollo and Vilson Serafim Júnior contributed exclusively to the antimicrobial activity studies. Moacir Fernandes de Godoy contributed to investigation and data interpretation. Margarete Teresa Gottardo de Almeida was responsible for the design of the antimicrobial activity studies, data acquisition, and data interpretation. José Geraldo Nery was responsible for conceptualization, formal analysis, data interpretation, supervision, writing of the original draft, review, and editing of the article, and project administration.

## Ethics Statement

The study was approved by the Research Ethics Committee of FAMERP (São José do Rio Preto Medical School), Brazil (Protocol CAAE 48358215.9.0000.5415), and was conducted in accordance with the ethical principles of the Declaration of Helsinki.

## Consent

The authors have nothing to report.

## Conflicts of Interest

The authors declare no conflicts of interest.

## Supporting information




Supporting Information is available from the publisher or from the corresponding author upon request. It includes additional physicochemical characterization data, extended figures, and supplementary datasets that support the analyses and conclusions presented in the main text.**Supporting File**: asia70755‐sup‐0001‐SuppMat.docx.

## Data Availability

The data supporting the findings of this study are available from the corresponding author upon reasonable request.
