# Supplementary material for: Nanometric Faujasite (FAU) Zeolite Ion‐Exchanged With Metal Ions for Hemostatic and Antimicrobial Applications: A Thromboelastographic and Microbiological Study
Source: Chem Asian J. 2026 Apr 29;21:e70755. doi: 10.1002/asia.70755 (PMC13129264; doi:10.1002/asia.70755)
Supplement: Supplementary file 1 — Supporting Information is available from the publisher or from the corresponding author upon request. It includes additional physicochemical characterization data, extended figures, and supplementary datasets that support the analyses and conclusions presented in the main text.Supporting File: asia70755‐sup‐0001‐SuppMat.docx. [file ASIA-21-e70755-s001.docx]

**Supporting Information**


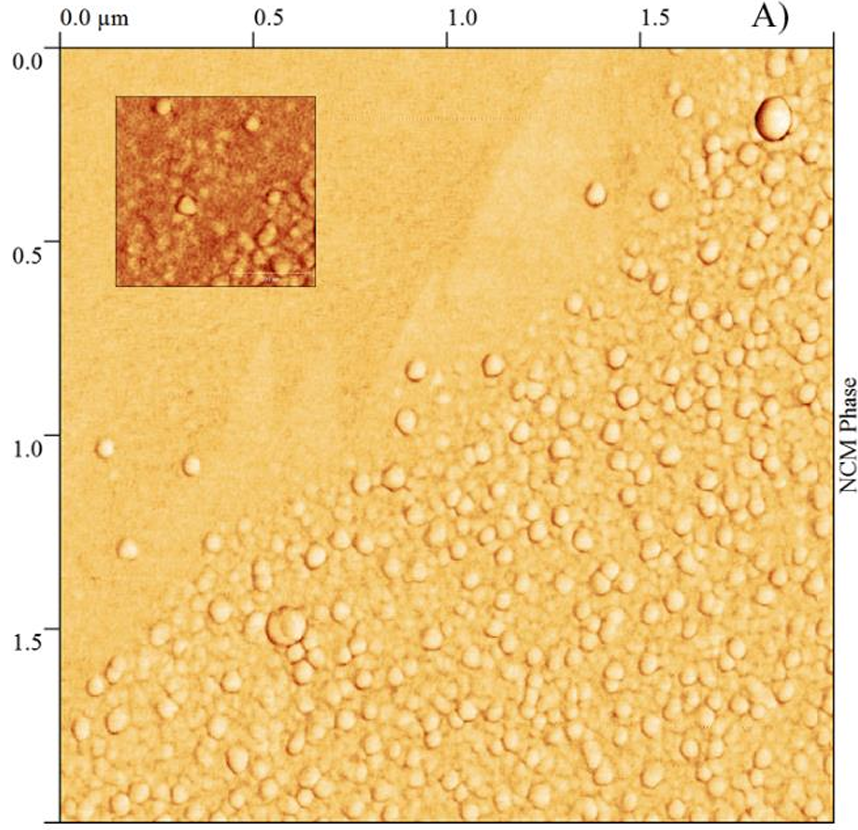


Figure SI1. Atomic Force Microscopy image of NanoFAU.


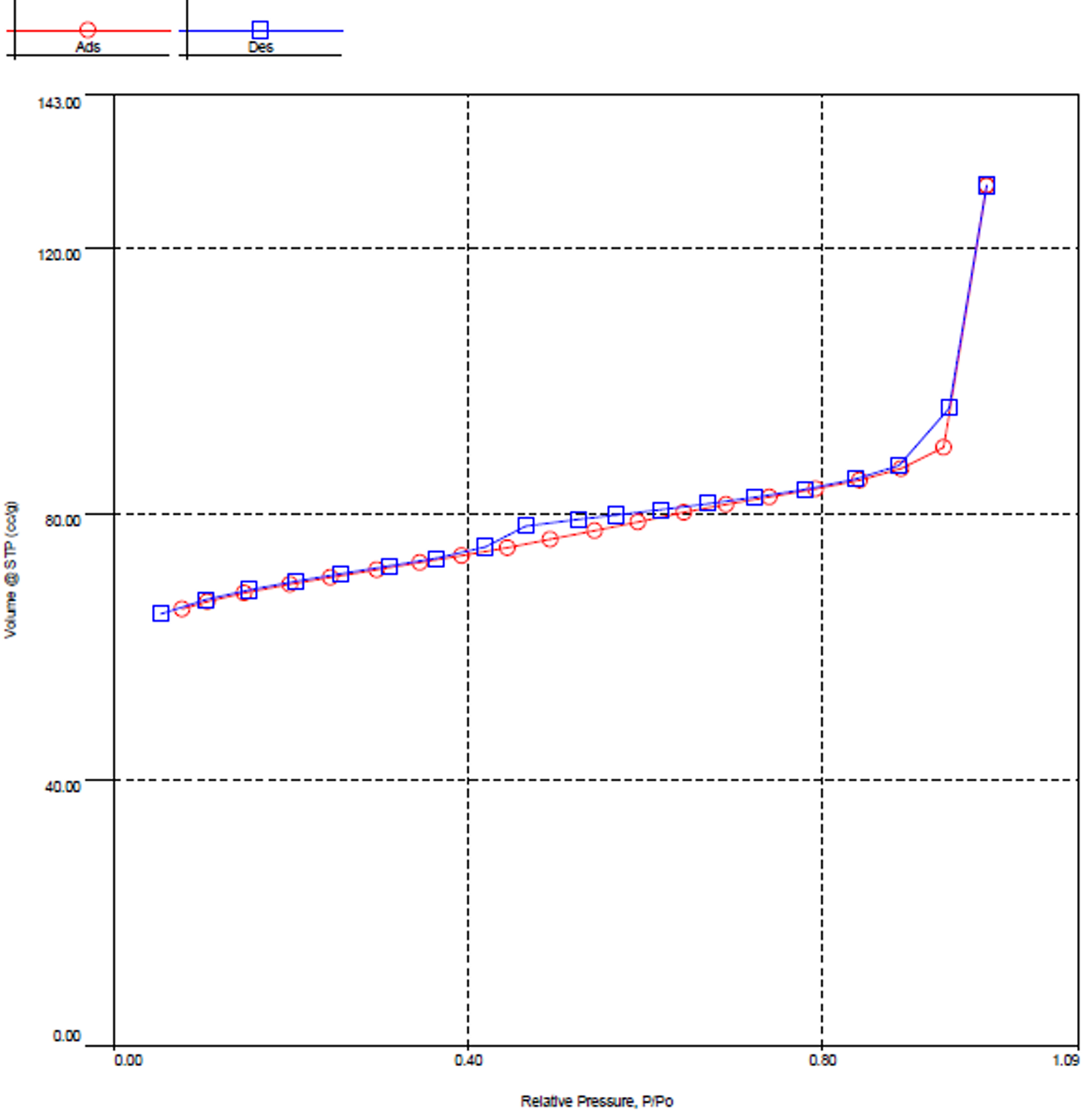


Figure SI2. Adsorption and desorption isotherms of nanometric Faujasite zeolite (NanoFAU).


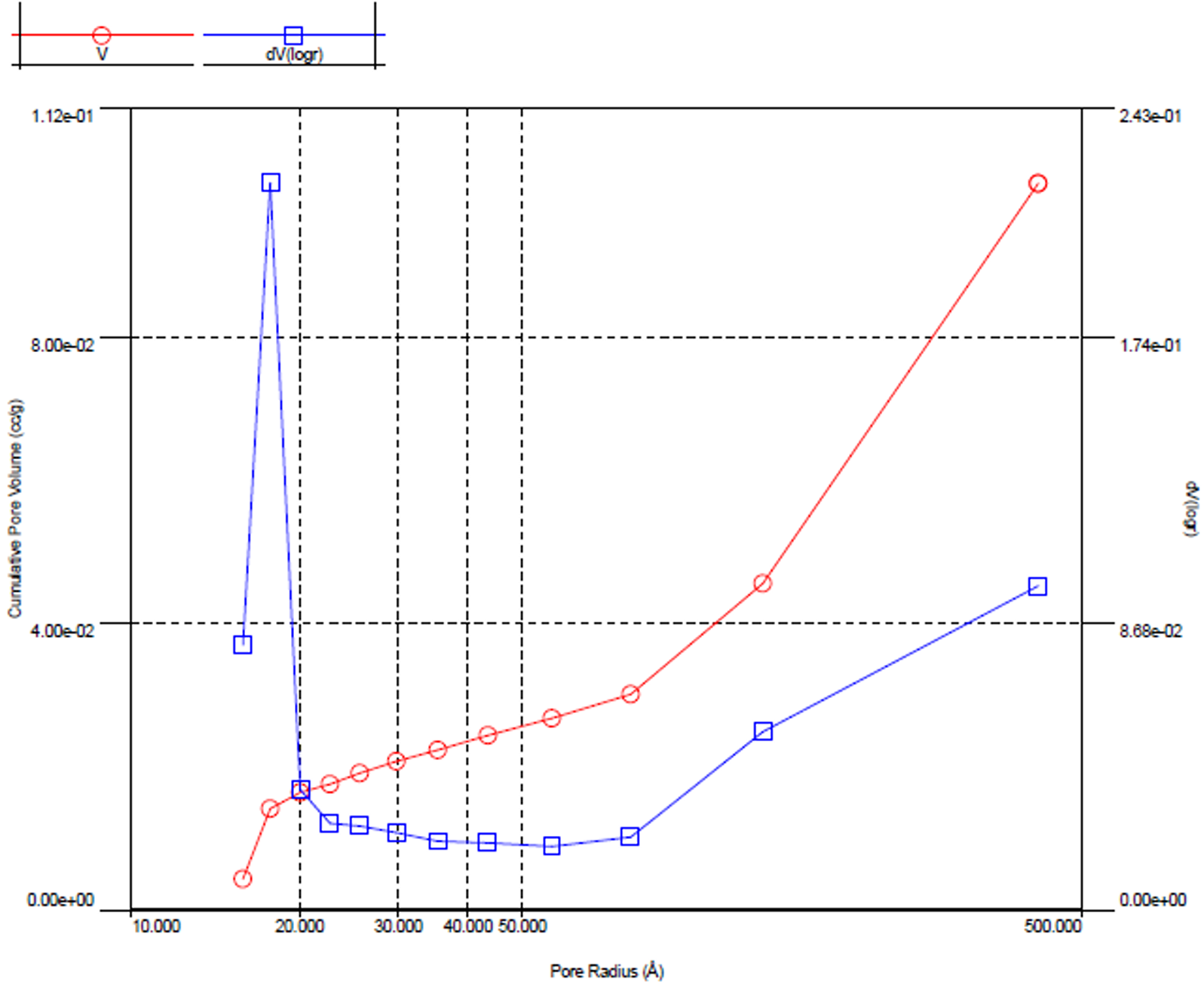


Figure SI3. Pore size distribution curve of the NanoFAU material.


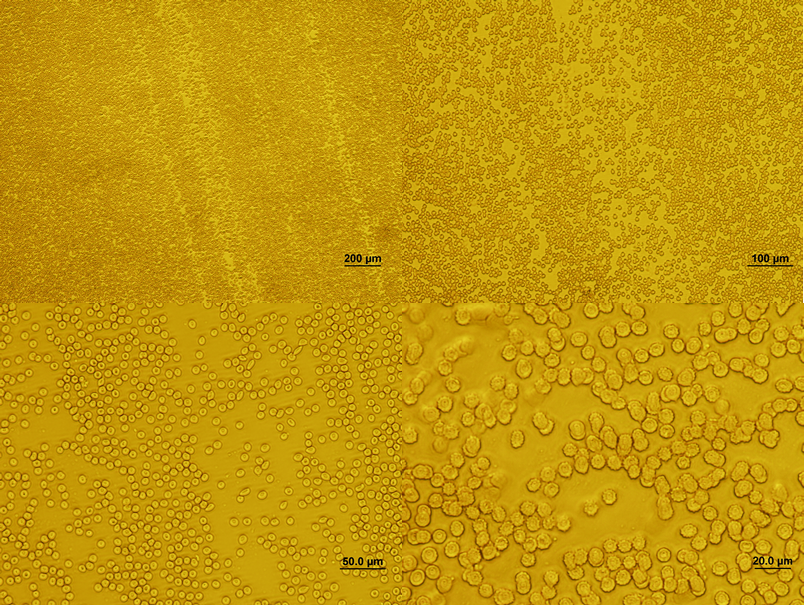


**Figure SI4.** Images of whole blood before the addition of zeolites obtained by phase-contrast microscopy


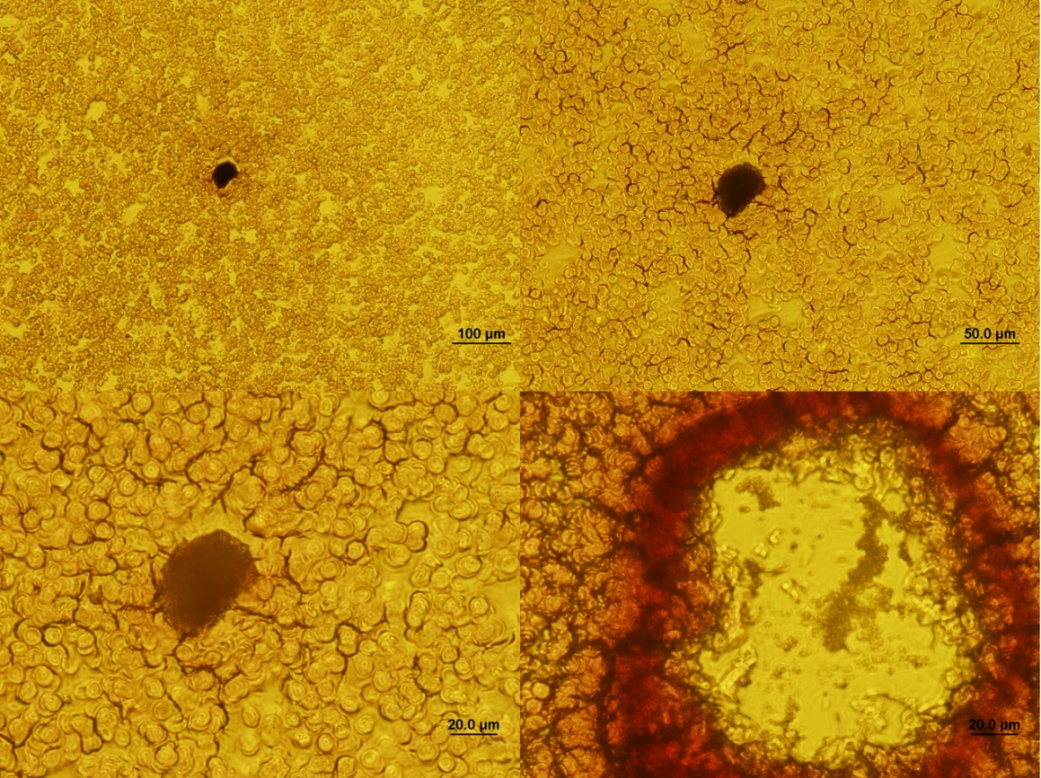


**Figure SI5.** Images of whole blood after the addition of NanoFAU zeolites obtained by phase-contrast microscopy.


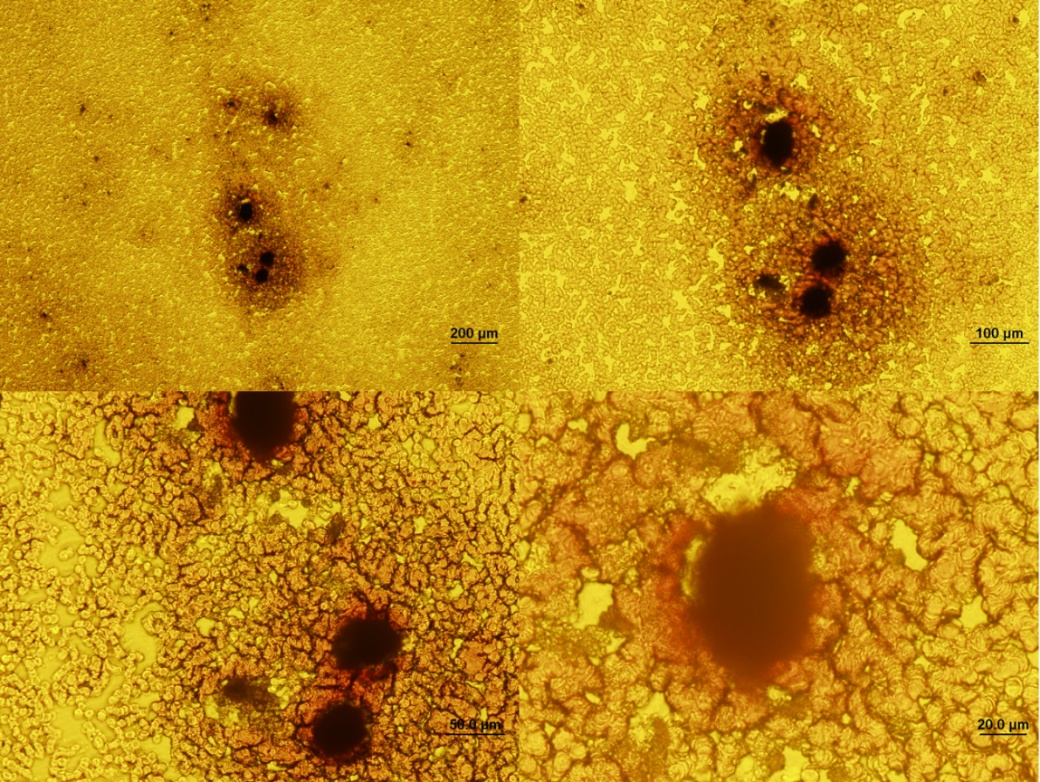


Figure SI6. Images of whole blood after the addition of NanoFAU-Ca zeolites obtained by phase-contrast microscopy.
